# Comparative proteomics in tall fescue to reveal underlying mechanisms for improving Photosystem II thermotolerance during heat stress memory

**DOI:** 10.1186/s12864-024-10580-z

**Published:** 2024-07-09

**Authors:** Guangyang Wang, Xiulei Wang, Dongli Li, Xuehe Yang, Tao Hu, Jinmin Fu

**Affiliations:** 1 School of Resources and Environmental Engineering, Ludong University, Yantai City, 264025 China; 2 Urban Management Bureau, Taiqian County, Puyang City, 457600 China; 3 State Key Laboratory of Herbage Improvement and Grassland Agro-ecosystems, College of Pastoral Agriculture Science and Technology, Lanzhou University, Lanzhou city, 730020 China

**Keywords:** Heat stress memory, Comparative proteomics, Photosystem II, Tall fescue, TMT labeling

## Abstract

**Background:**

The escalating impacts of global warming intensify the detrimental effects of heat stress on crop growth and yield. Among the earliest and most vulnerable sites of damage is Photosystem II (PSII). Plants exposed to recurring high temperatures develop heat stress memory, a phenomenon that enables them to retain information from previous stress events to better cope with subsequent one. Understanding the components and regulatory networks associated with heat stress memory is crucial for the development of heat-resistant crops.

**Results:**

Physiological assays revealed that heat priming (HP) enabled tall fescue to possess higher Photosystem II photochemical activity when subjected to trigger stress. To investigate the underlying mechanisms of heat stress memory, we performed comparative proteomic analyses on tall fescue leaves at S0 (control), R4 (primed), and S5 (triggering), using an integrated approach of Tandem Mass Tag (TMT) labeling and Liquid Chromatography-Mass Spectrometry. A total of 3,851 proteins were detected, with quantitative information available for 3,835 proteins. Among these, we identified 1,423 differentially abundant proteins (DAPs), including 526 proteins that were classified as Heat Stress Memory Proteins (HSMPs). GO and KEGG enrichment analyses revealed that the HSMPs were primarily associated with the “autophagy” in R4 and with “PSII repair”, “HSP binding”, and “peptidase activity” in S5. Notably, we identified 7 chloroplast-localized HSMPs (HSP21, DJC77, EGY3, LHCA4, LQY1, PSBR and DEGP8, R4/S0 > 1.2, S5/S0 > 1.2), which were considered to be effectors linked to PSII heat stress memory, predominantly in cluster 4. Protein-protein interaction (PPI) analysis indicated that the ubiquitin-proteasome system, with key nodes at UPL3, RAD23b, and UCH3, might play a role in the selective retention of memory effectors in the R4 stage. Furthermore, we conducted RT-qPCR validation on 12 genes, and the results showed that in comparison to the S5 stage, the R4 stage exhibited reduced consistency between transcript and protein levels, providing additional evidence for post-transcriptional regulation in R4.

**Conclusions:**

These findings provide valuable insights into the establishment of heat stress memory under recurring high-temperature episodes and offer a conceptual framework for breeding thermotolerant crops with improved PSII functionality.

**Supplementary Information:**

The online version contains supplementary material available at 10.1186/s12864-024-10580-z.

## Background

Spurred by global warming, the frequency and severity of extreme weather events (e.g., extreme high temperature) keep rising, which gravely threaten the plant growth and cause devastating damage to crop productivity [1]. Tall fescue (*Festuca arundinacea* Schreb.), a cool-season grass, thrives in temperate zones as a primarily used forage or turfgrass. Its most efficient growth temperatures for above-ground parts lie within a range of 15–24 °C. Temperatures exceeding 30 °C were observed to provoke a pronounced stress response, leading to a yellow, withered plant, and ultimately resulting in plant death [2]. Its adaptation to cooler climates means that it can exhibit a more pronounced stress response when exposed to high temperatures, providing a distinct model to investigate the genetic and cellular pathways involved in heat stress.

Sophisticated regulatory networks are equipped to withstand heat stress (HS). Upon HS, the protein unfolding [3] and reactive oxygen species (ROS) burst [4] are primarily triggered and leads to the intracellular homeostasis disturbances. They then, along with calcium spike, initiate a sequence of heat stress response (HSR) [5]. The principle functional HSR genes are heat shock protein *(HSP)* and ROS scavengers like superoxide dismutase (*SOD*), catalase (*CAT*), ascorbate peroxidase (*APX*) [2]. A host of transcriptional regulators, led by heat shock transcription factor A1s (HSFA1s), are involved in HSR, such as the *dehydration- responsive element binding protein 2 A* (*DREB2A*), *HSFA2*, *HSFB* and *HSFA7s* [5, 6]. Furthermore, post-translational regulations modify DREB2A and HSFA1 activities through SUMOylation [7], ubiquitination [8], phosphorylation [9, 10] and protein-protein interactions (PPI) [11, 12]. In addition, microRNAs (miRNAs) are closely associated with HSR. For instance, miR398 is induced by HSFA1 and targeted genes coding ROS-scavenging enzymes [13]. In turn, excessive ROS further induced HSFA1, forming positive feedback. However, rapid fluctuations of ambient temperature over time can lead to repeated heat stress, rendering these mechanisms insufficient.

Plants have developed the ability to get into a primed state after the exposure to a past heat shock, readying themselves for subsequent episodes, usually in the form of a faster and stronger response. This process is referred to as acquired heat stress resistance and the ability to retain the primed state over time is classified as HS memory [14, 15]. Over the years, extensive research has focused on identifying the components of heat shock memory, categorising them broadly into effectors and regulators [16], termed as heat stress memory proteins (HSMPs) in this study. The effectors are considered to be the physical substances induced by priming, regulate the next stress manifestations. The commonest being heat shock proteins, like HSP101 and HSP21. While, the duration of effectors is controlled by the regulators. For instance, HSP101 decay after priming is slowed by the HSP101-HSA32 positive feedback loop [17], under the control of HSFA2/HSFA3. Or HSP21 abundance is positively regulated by ROF1 and HLP1 [18–20].

Importantly, information storage from past environmental disturbances is linked with epigenetic mechanisms, which influence gene transcription by regulating DNA accessibility to transcriptional machinery [21]. The altered epigenetic characteristics reported in HS memory, including but not limited to histone modifications (e.g., H3K4me3, and H3K27me3) and nucleosome remodeling [15]. H3K4me3, a mark for transcriptional activation, contributes significantly to HS memory [22, 23]. The establishment of H3K4me3 depends on HSFA2 [24], which was in connection with the recruitment of the histone methyltransferase, Compass-like complex. H3K27me3 is associated with transcriptional suppression [25]. Histone demethylase JMJ is implicated in HS memory by maintaining H3K27me3 demethylation of *HSP* genes, for instance, the HSFA2-REF6 regulatory loop [26]. The chromatin remodeling proteins complex (FGT1-BRM-CHR11/ CHR17) is also reported to be involved in HS memory [27]. Another protein, BRU1, is responsible for the sustained transcriptional activation of effector genes in HS memory [28], which may be related to its ability to faithfully inherit chromatin status during DNA replication and cell division [29]. The above factors are considered to be HS memory maintainers. Note that the memory retention and loss during recovery are finely balanced between effectors accumulation and decay, the autophagy mechanism is defined as a memory eraser. For example, NBR1 and Ftsh6 respectively mediated the degradation of HSP90.1 and HSP21 [30, 31]. Despite the progress made in this field, a comprehensive molecular regulatory network of HS memory components remains an ongoing area of study.

Photosynthesis-associated processes are widely believed to be very susceptible to heat stress [32–34]. In which a tight coupling of light and dark reactions is disturbed first. This is owing to the photoinduced electron transport activity is largely affected by light intensity, whereas the Calvin-Benson cycle is temperature-sensitive, e.g., the activity of rubisco activase (RCA) has been observed to be inhibited at moderate temperature elevation [35]. This leads to an imbalance in ATP and NADPH production and consumption, causing an over-reduction of the electron transport chain, which in turn, results in reactive oxygen species (ROS) production [36]. The primary target of photo-oxidative damage is D1 protein [37]. To cope with environmental disturbances, extensive adjustments take place in chloroplast to ensure the normal operation of the photosynthetic apparatus. Multiple energy overflow pathways such as photorespiration, cyclic electron transfer and non-photochemical quenching (NPQ) avoid over-reduction of electron transport chain [38, 39]. Additionally, A range of enzymatic (e.g., SOD, POD, CAT) and non-enzymatic antioxidants (e.g., AsA-GSH cycle) limits the ROS content [40]. The protein phosphorylase (e.g., STN7/8) [41], protease (e.g., Deg, FtsH) [37] and chaperone proteins (e.g., HSP20) [42] are involved in D1 protein turnover. The existence of HS memory components in the vast and complex photosynthetic reprogramming under recurrent HS is yet to be explored.

As crucial constituents and facilitators of physiological functions, proteins represent the material bedrock on which these processes depend. Consequently, evaluating alterations in intracellular protein composition serves as an expedient strategy for understanding the mechanisms underpinning physiological responses. In this work, we implemented a tandem mass tag (TMT) label-based quantitative proteomics methodology, enhanced by liquid chromatography-tandem mass spectrometry (LC-MS/MS), to investigate differentially abundant proteins in leaves at three stages (Control-S0, Primed-R4, Triggering-S5), with a fold-change threshold set at 1.2. R4 signifies a critical phase in initiating HS memory, marked by the shift from acute stress response to the activation of enduring adaptive mechanisms. Similarly, S5 is instrumental in examining the perpetuation and strengthening of heat priming-induced adaptations. This research potentially provides a theoretical foundation for the heat-resistance breeding of tall fescue and other crops by identify the effectors and regulators of HS memory during photosynthesis.

## Results

### Heat priming improved PSII photochemical performance against following heat stress

**Fig. 1:**
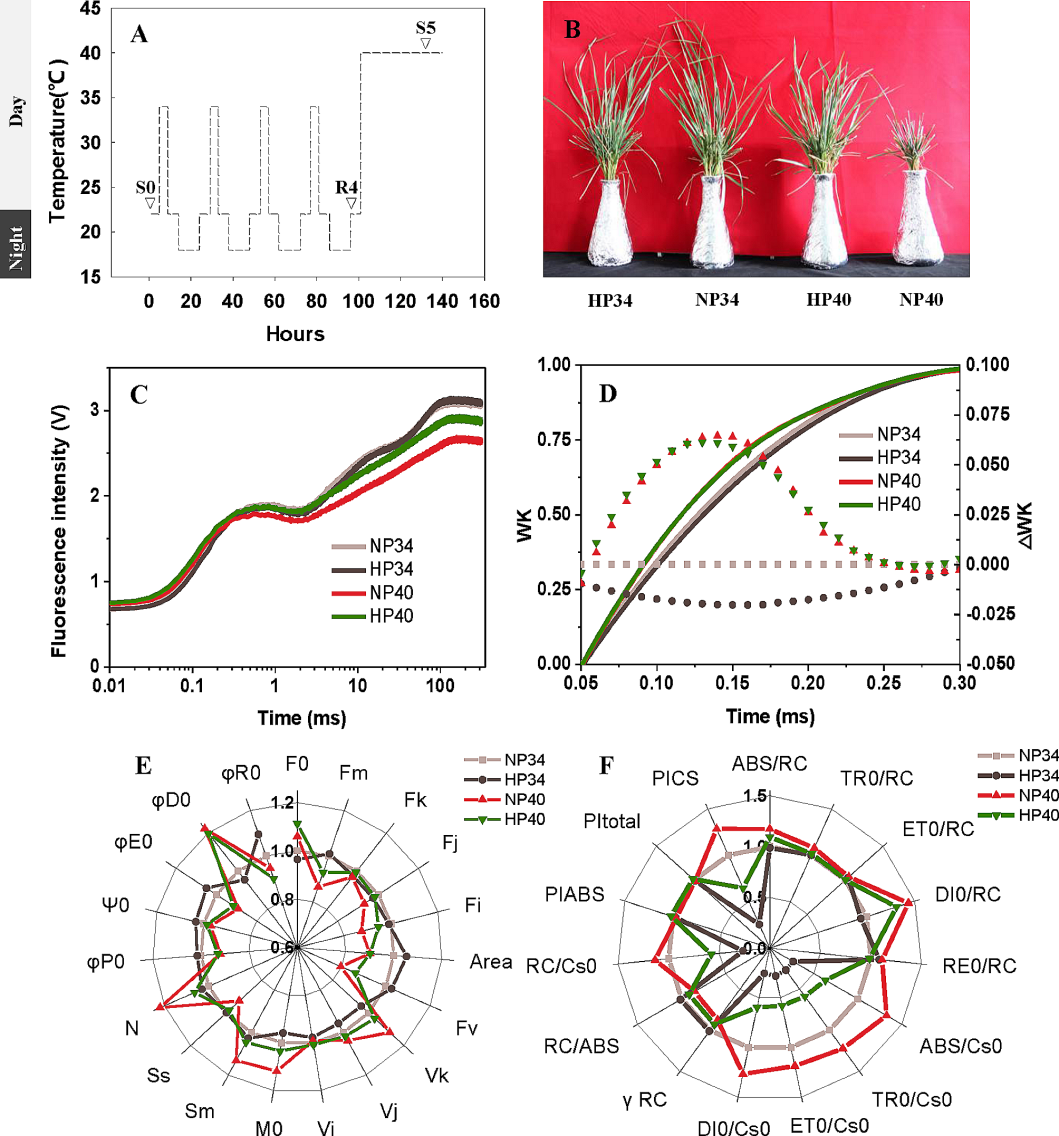
**PSII photochemical activity of heat primed tall fescue under triggering stress. (A)** The timeline of the diurnal temperature shifts incorporated in the heat priming protocol, artificially segmented into three stages. **Control before ‘Priming’, green box**: plants grew in optimum temperature 22/18°C (day/night) for one week to acclimate to the environmental conditions of the growth chambers. **Multiple ‘priming’ stimulus, yellow box**: plants were subject to continuous sub-high temperature (34 °C) stimulus 4 h at noon, which is repeated four times. **Triggering stress, red box**: primed plants endure high temperature (40 °C) stress over 36 h. The protocol commences at 0 h. All phases adhere to a 14/10 (day/night) photoperiod, signified by grey (day) and black (night) boxes near the y-axis. **The triangle** represented the sampling time (before heat priming, primed, triggering) for proteomic analysis, assigned as **S0**, **R4**, **S5** respectively. **(B)** Completed the **heat priming (HP)** protocol shown in Fig. 1A yellow box, HP34 and HP40 group were retained at 34 ^o^C and 40 ^o^C respectively for 36 h, and the **non-priming (NP)** groups, serves as the control. Images of the tall fescue phenotype are captured following a week of recovery. **(C)** Polyphasic rise of chlorophyll fluorescence transients under four treatments. There were five biological replicates in present study. **(D)** Energetic connectivity of PSII, W_k_= (F_t_-F_o_)/(F_K_-F_o_), ΔW_K_ =W_K treatment_-W_K NP34_. **(E, F)** Radar plots of fluorescence transient parameters derived by JIP-test. NP34 was defined as 1. Detailed notes and data for the photochemical parameters in the radar plots were listed in Table S1. Abbreviations: HP34, HP40; Tall fescue previously strengthened through heat priming and exposed once more to a heat shock of either 34–40 °C. NP34, NP40; Tall fescue without heat priming was directly subjected to heat shock at 34 ℃, 40 ℃, respectively

As shown in Fig. 1B, pre-experienced heat priming (HP), HP40 survived from deadly heat shock. However, growth inhibition was observed in HP34 when compared with NP34. It suggested HP was actually a mild heat injury, however, from which the tall fescue acquired stronger heat stress resistance. To further elucidate the protective role of HP, present research investigated the activity of photosynthetic electron transfer by JIP-tests under HP conditions. The result showed NP40 had a positive L-band value (Fig. 1D) and alongside pronounced decreases in F_V_, RC/ABS and φE_0_ (Fig. 1C, E, F, **Table ****S1**), indicating the suppression of the energy connectivity among subunits of PSII, Q_A_-reducing reaction center (RC) per PSII antenna Chl, and quantum yield of electron transport by HS. This partial deactivation of RCs led to the increases of ABS/RC, TR_0_/RC and RE_0_/RC, which implicated an elevated risk of light damage to RCs. Intriguingly, these harmful effects were notably mitigated by heat priming. For instance, HP34 showed a negative L-band (Fig. 1D). F_V_, RC/ABS, φE_0_, ABS/RC, TR_0_/RC and RE_0_/RC were partly restored in HP40 compared to NP40 (Fig. 1C, E, F, **Table ****S1**). Given the augmented photochemical function of PSII under heat stress after heat priming, a differential proteomic analysis was conducted at three stages (before heat priming/S0, primed/R4, triggering/S5) (Fig. 1A).

### iTRAQ analysis and profile of proteins altered by heat priming

**Fig. 2:**
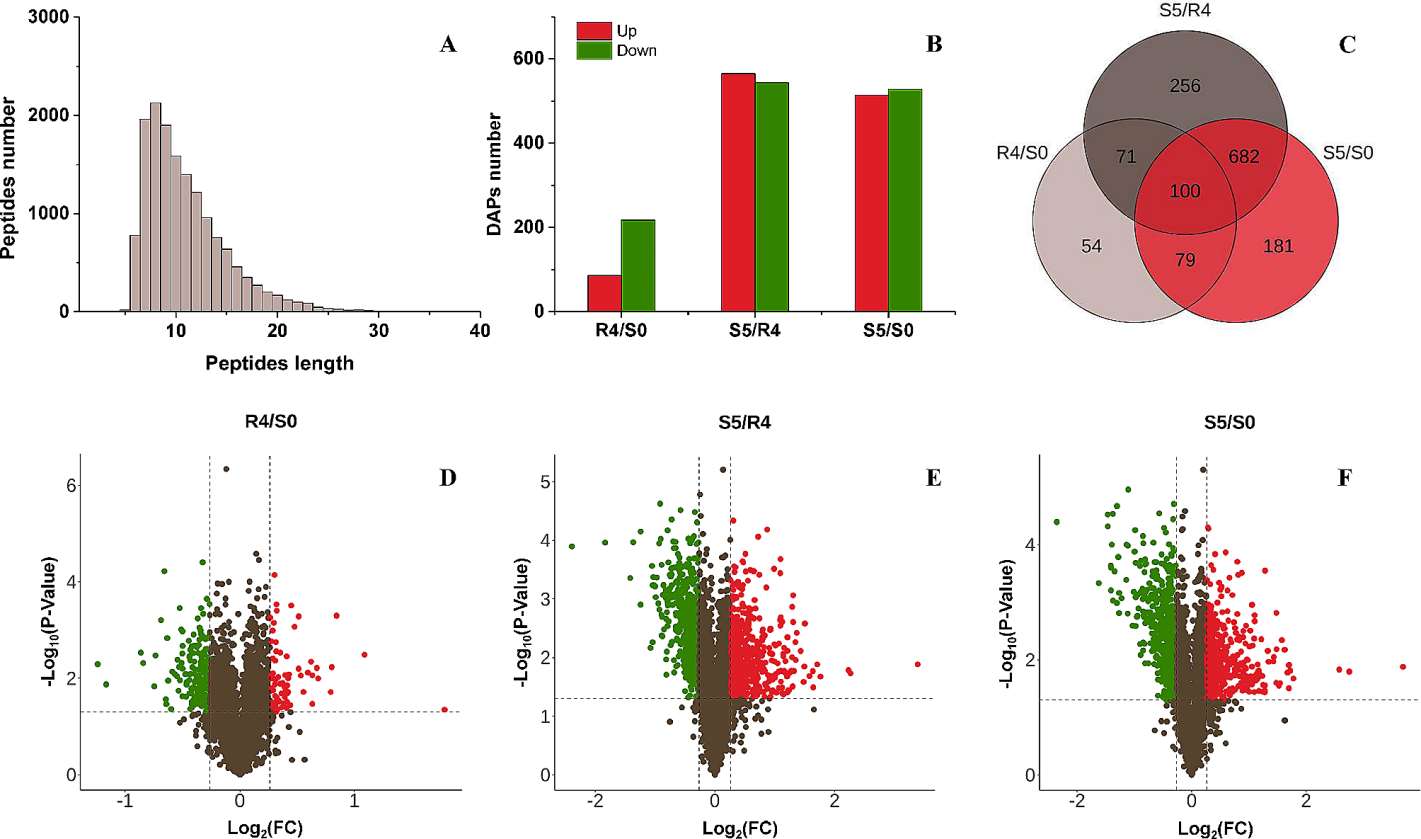
**A general overview of DAPs identification in three time phases. (A)** Distribution of peptide lengths. **(B)** The number of upward and downward DAPs ( Fold changes > 1.2, *p* < 0.05). **(C)** Venn Diagrams of DAPs. Every circle in the figure represents a comparison group, where the numbers in the overlapping part indicate the number of DAPs shared between the two or three, while numbers in the non-overlapping sections represent the number of specific DAPs in each group. Accordingly, the DAPs in R4/S0 and S5/S0 were classified into three categories, and then by going through de-redundancy and conditional screening, we identified 125 R4-specific DAPs (R4/S0 > 1.2, S5/S0 < 1.2), 135 HP-retained DAPs (R4/S0 > 1.2, S5/S0 > 1.2) and 266 HP-induced DAPs (R4/S0 < 1.2, S5/R4 > 1.44). Volcano plot of DAPs in R4/S0 **(D)**, S5/R4 **(E)** and S5/S0 **(F)** were displayed. Horizontal coordinate represent fold changes (FC) in protein abundance (log2 value) and vertical coordinate represent P-values (-log10 value). Brown dots indicate proteins that were not differentially expressed; red and green dots represent significantly up- and down-regulated proteins, respectively

Intrigued by the noted variations in phenotype and photochemical activities, a comparative proteomic analysis was performed employing TMT labeling and liquid chromatography-tandem mass spectrometry (LC-MS/MS). Leaf samples from tall fescue at S0 (control), R4 (primed), and S5 (triggering) time points were used as test materials with three biological replicates established. The study aimed to glean an understanding of the underlying mechanisms invoked by heat priming (HP) to create such observable differences. Emphasis was placed on the proteins retained at R4 or induced at S5 as a means to elucidate the physical basis of heat stress (HS) memory maintenance or to examine the reasons behind improved HS resistance. Following rigorous quality control filtration, 3851 distinct proteins were identified from top-quality 15,288 peptides, with 3835 proteins showing quantitative data across all three stages. A total of 1423 differentially abundant proteins (DAPs) (P-value < 0.05, Fold Change (FC) > 1.2 or FC < 5/6) were ultimately uncovered (see **Additional file2: Table ****S2**for corresponding DAPs). Expectedly, most peptide length was distributed within the 6–20 amino acid residues range (Fig. 2A), which aligns with peptide fragments yielded by trypsin digestion, confirming the efficacy of the preliminary treatment in complying with the test requirements. Moreover, the hierarchical cluster analysis among each time point’s replicates displayed high repeatability (**Additional file3: Fig ****S1**).

Upon treatment with HP, 304 differentially abundance proteins (DAPs) were identified in the leaves of R4 when compared to S0, among which 86 were up-regulated and 218 were down-regulated (Fig. 2B). In the subsequent HS period (S5), a comparison with S0 revealed 1042 DAPs, with 514 being up-regulated and 528 being down-regulated **(**Fig. 2B). A comparison of protein abundance between R4 and S5 yielded 1109 DAPs, comprising 565 up-regulated and 544 down-regulated proteins (Fig. 2B). The volcano plot of the DAPs of R4/S0, S5/R4 and S5/S0 is shown in Fig. 2D, E, F. DAPs that were generally or specifically targeted by HP were determined by overlapping the DAPs in R4/S0, S5/R4, and S5/S0 (Fig. 2C). There were 125 DAPs (123 after de-redundancy) that exhibited differential abundance in R4/S0 but not in S5/S0 (**Additional file4: Table ****S3**). Among these, 28 were up-regulated and 94 were down-regulated and were defined as R4-specific DAPs. We propose these were involved in thermal injury repair from HP, and in the reorganization of regulatory components for the next HS. In addition, we observed 179 DAPs shared between R4/S0 and S5/S0. Specific focus was given to proteins that were synchronously up- and down-regulated in both R4/S0 and S5/S0. We postulate that these proteins, which include 45 up-regulated and 90 down-regulated DAPs (defined as HP-retained DAPs, **Table ****S3**), have a longer half-life after HS. More specifically, these are potential functional elements that underlie the HS memory. Furthermore, 863 DAPs (over half of the total DAPs) displayed differential abundance only in S5. These proteins quickly returned to initial levels resembling S0 following HS withdrawal. We hypothesized that these protein level changes align with the S4/R4 > 1.2 provision. Consequently, DAPs with the S5/R4 > 1.44 feature, which is an increase from S5/R4 > 1.2*1.2, are induced by HP and have a stronger response than the last HS (S4). Finally, 266 proteins were identified as candidate HP-induced DAPs (**Table ****S3**).

**Fig. 3:**
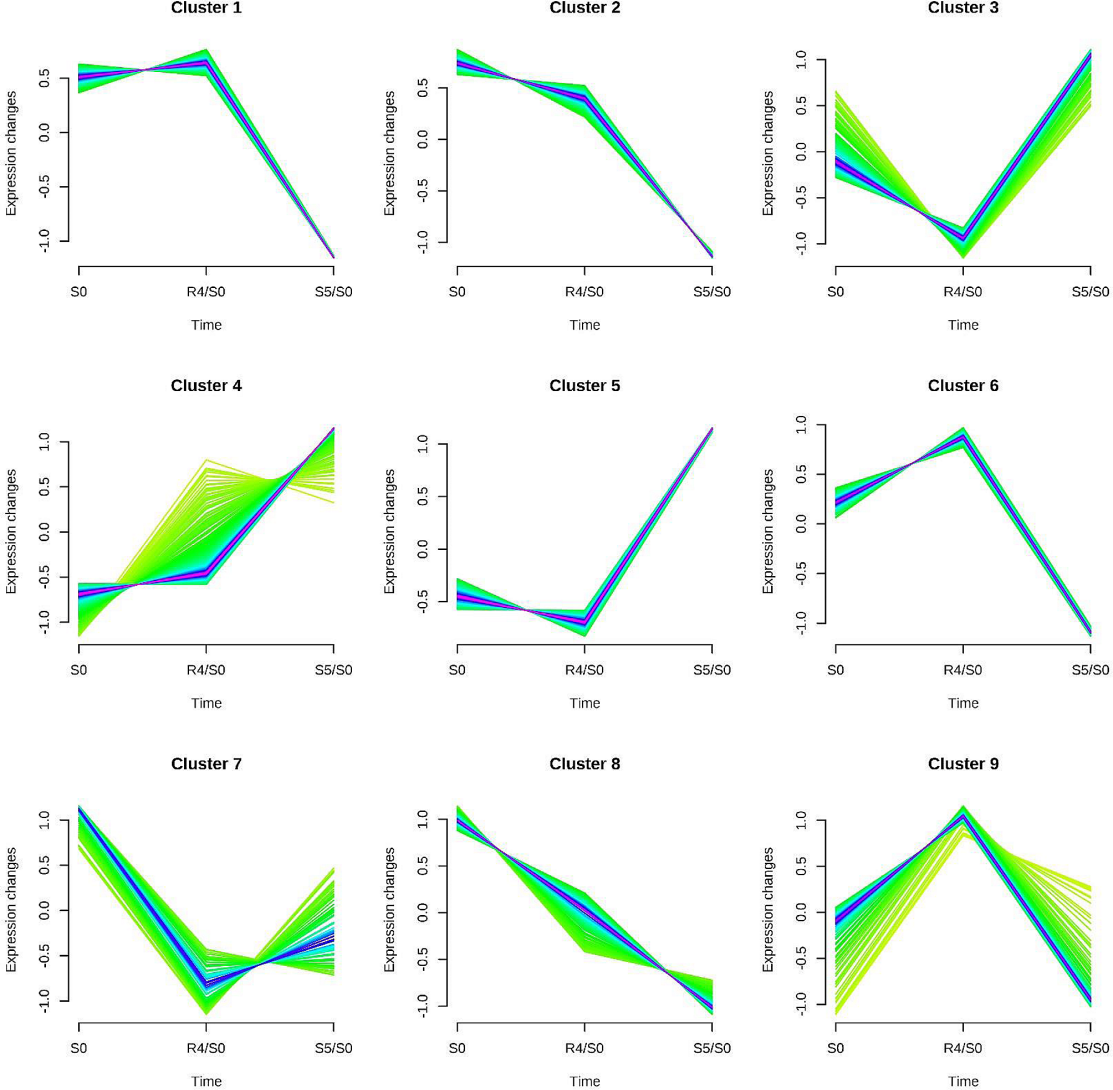
**Soft clustering based on temporal dynamics of DAPs profiles.** A total of 1421 DAPs underwent soft clustering via Mfuzz’s fuzzy C-means algorithm with the number of clusters predetermined at 9. Missing values were algorithmically replaced by the mean, however, data rows were omitted if the missing value count exceeded 66% of the total

Based on protein abundance changes at three time points, a fuzzy c-means algorithm (Mfuzz software) was used to perform soft clustering of all DAPs (1421 after de-redundancy). In total, 9 clusters were divided, Cluster 1 (157 DAPs), Cluster 2 (137 DAPs), Cluster 3 (176 DAPs), Cluster 4 (295 DAPs), Cluster 5 (200 DAPs), Cluster 6 (118 DAPs), Cluster 7 (84 DAPs), Cluster 8 (143 DAPs), Cluster 9 (111 DAPs), as illustrated in Fig. 3. R4-specific DAPs were predominantly categorized in cluster 3 (46 DAPs, 37.70%, down-regulated), cluster 7 (48 DAPs, 39.34%, down-regulated) and cluster 9 (28 DAPs, 22.95%, up-regulated) (Fig. 3, **Table ****S3**). Within these proteins, both effectors (within chloroplasts) and regulators had been particularly noted. Interestingly, up-regulated DAPs are almost always effectors, while regulators are only found in down-regulated DAPs, e.g., E3 ubiquitin-protein ligase UPL3 (comp_71_120270_c0_seq1). HP-retained DAPs were primarily categorized in cluster 4 (41 DAPs, 30.37%, up-regulated), cluster 7 (31 DAPs, 22.96%, down-regulated) and cluster 8 (56 DAPs, 41.48%, down-regulated) (Fig. 3, **Table ****S3**). The data suggested DAPs with delayed recovery after HP and can carry over to the next stress are mostly down-regulated. The up-regulated DAPs, particularly those localized within the chloroplasts, drew our attention. A heat shock protein HSP21 (comp_71_109284 _c0_seq3) maintained the highest abundance at R4. More, the DAPs induced by HP were categorized specific clusters, with the ones up-regulated primarily in clusters 3 (12 DAPs, 4.51%), 4 (76 DAPs, 28.57%) and 5 (74 DAPs, 27.82%); and the ones down-regulated in clusters 1 (44 DAPs, 16.54%), 2 (44 DAPs, 16.54%) and 6 (30 DAPs, 11.28%), as depicted in Fig. 3 and **Table ****S3**.

### GO enrichment analysis of DAPs

**Fig. 4:**
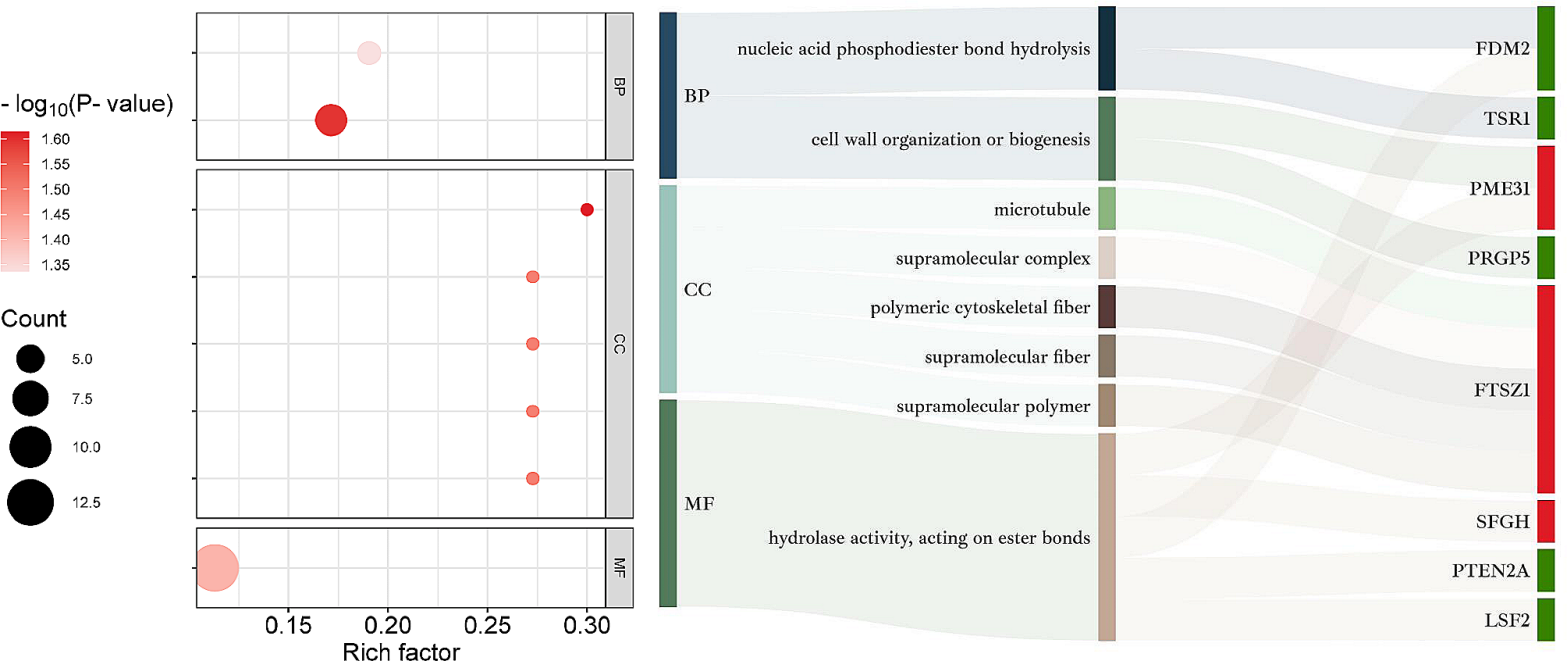
**GO enrichment analysis of DAPs in R4.** DAPs in R4/S0 were categorized into three groups: molecular function (MF), biological processes (BP), and cellular components (CC). Gene Ontology (GO) terms that include HSMPs were only depicted in the left half of Fig. 4. In the bubble diagram, the size and color of each dot correspond to the number of DAPs under each GO term and the significance of the enrichment, respectively. The ‘rich factor’ is a ratio that compares the number of DAPs in a specific GO term to the total number of proteins in that GO term. The right half of Fig. 4 showcases a Sankey diagram mirroring the GO terms, along with the associated HSMPs. Dark red or green colors next to the protein names represent up-regulation or down-regulation of DAPs specific to the R4, respectively

**Fig. 5:**
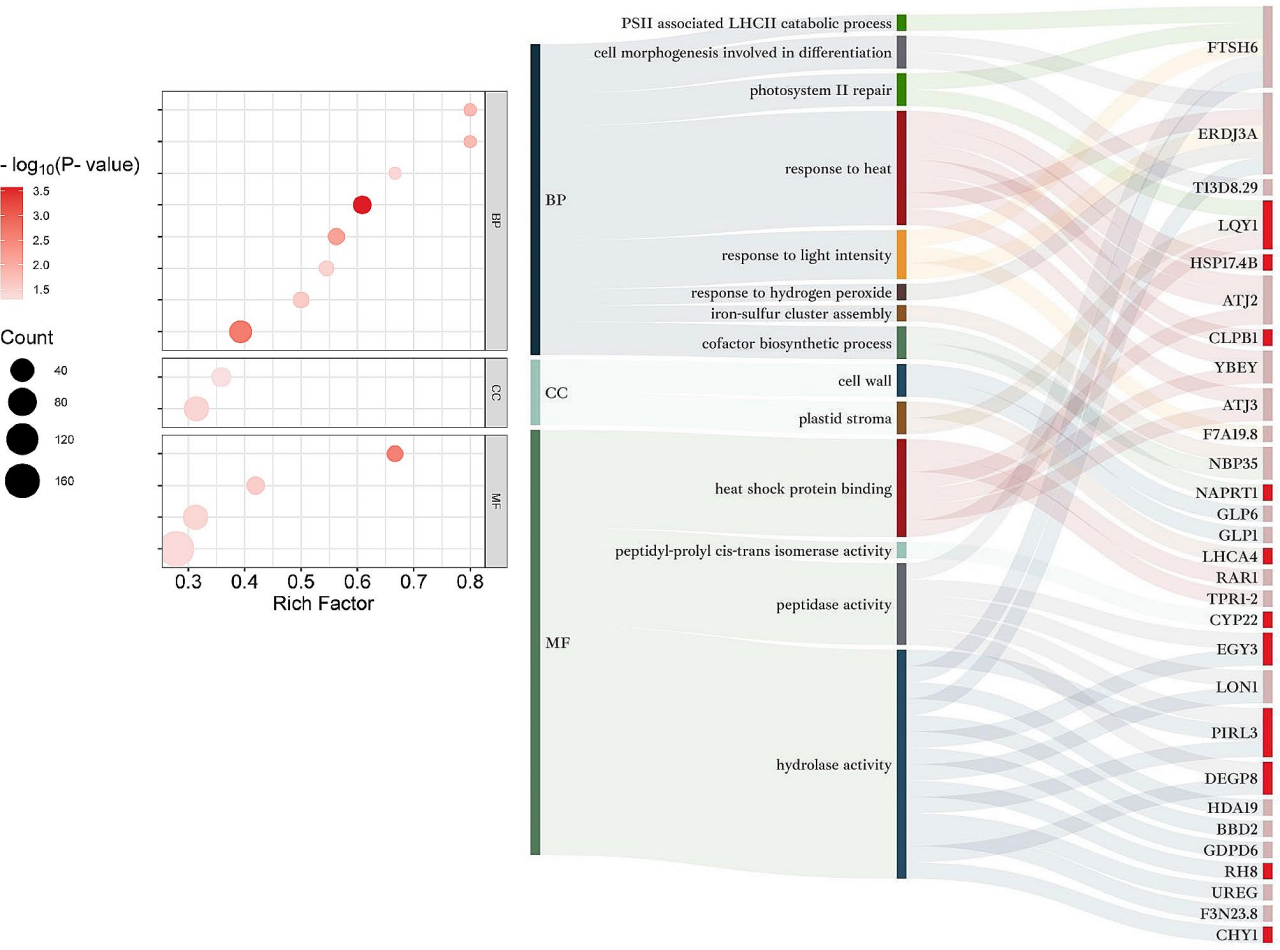
**GO enrichment analysis of DAPs in S5** As shown in Fig. 4, the Gene Ontology (GO) enrichment analysis was divided into three categories: Biological Process (BP), Cellular Component (CC), and Molecular Function (MF). GO terms containing HSMPs were presented in the bubble plots. The bubble diameter and color intensity represented the number of DAPs affiliated with each GO term, and their enrichment significance, respectively. The rich factor was characterized as the ratio of DAPs in a specific GO term against the total number of proteins within that term. Adjacent to the bubble chart was the Sankey diagram, illustrating corresponding HSMPs and GO term identifications in the same row. The segments represented by deep and pale red hues after protein names indicated DAPs retained and induced by HP, respectively

The biological information of DAPs was annotated using GO enrichment analysis which classified them into three categories: Biological Processes (BP), Cellular Components (CC) and Molecular Function (MF). Proteins related to heat stress memory (HSMPs) in GO terms were displayed in Figs. 4 and 5; Table 1.

**Table 1:** Differentially abundant proteins(DAPs)related to heat stress memory in this work

	Accession	R4/S0	S5/S0	Name	annotation
**R4-specific -Effectors**	comp_159_125998_c0_seq1	1.73	1.19	*PME31*	Pectin methylesterase 31
	comp_71_113460_c0_seq1	1.23	0.84	**MAP1B**	Methionine aminopeptidase 1B
	comp_71_109193_c0_seq1	1.22	1.04	*SFGH*	S-formylglutathione hydrolase
	comp_71_106493_c0_seq1	1.21	1.03	*FTSZ1*	Homolog of bacterial cytokinesis Z-ring protein FTSZ 1–1
**R4-specific Regulators**	comp_159_128959_c0_seq3	0.83	1.10	**Y14**	RNA-binding protein
	comp_71_121776_c1_seq1	0.83	0.96	CAM7	Calmodulin-7
	comp_71_101711_c0_seq1	0.83	0.95	**RPS25B**	Small ribosomal subunit protein
	comp_71_118763_c0_seq5	0.81	0.91	**RAD23B**	Rad23 UV excision repair protein family
	comp_71_118855_c0_seq3	0.81	1.07	**BTF3**	Basic transcription factor 3
	comp_71_117432_c0_seq3	0.80	1.12	**T25O11.11**	Eukaryotic translation initiation factor 3 subunit J
	comp_71_122344_c0_seq1	0.79	0.99	*FDM2*	Factor of DNA methylation 2
	comp_159_132820_c0_seq3	0.78	0.94	**RNU1**	U1 small nuclear ribonucleoprotein 70 kDa
	comp_71_124939_c0_seq1	0.76	1.14	**RGGA**	RGG repeats nuclear RNA binding protein A
	comp_159_131602_c0_seq8	0.76	0.83	**RPS30A**	Small ribosomal subunit protein
	comp_159_125448_c0_seq6	0.74	0.93	**UCH3**	Ubiquitin C-terminal hydrolase 3 (UCH3)
	comp_71_120270_c0_seq1	0.73	0.99	UPL3	E3 ubiquitin-protein ligase
**HP-retained** **-Effectors**	comp_71_109284_c0_seq3	1.80	2.80	**HSP21**	Heat shock protein 20, chloroplastic
	comp_159_132147_c0_seq1	1.55	1.77	*EGY3*	Ethylene-dependent gravitropism-deficient and yellow-green-like 3
	comp_159_125549_c1_seq5	1.45	3.43	**HSP70-1**	mediator of RNA polymerase II transcription subunit 37e
	comp_159_119519_c1_seq1	1.43	1.43	*LHCA4*	Light-harvesting chlorophyll-protein complex I subunit A4
	comp_159_133513_c1_seq23	1.36	1.26	**LQY1**	DnaJ/Hsp40 cysteine-rich domain superfamily protein
	comp_159_126276_c0_seq8	1.33	1.66	*NAPRT1*	Nicotinate phosphoribosyltransferase 1
	comp_159_127534_c1_seq6	1.31	1.53	**HOP1**	Stress-induced-phosphoprotein 1
	comp_159_116978_c1_seq5	1.288586	6.75	*HSP17.4B*	17.4 kDa class III heat shock protein
	comp_71_118005_c1_seq7	1.28	1.34	*DEGP8*	Trypsin family protein with PDZ domain; Encodes DEG8
	comp_71_123489_c0_seq2	1.25	1.92	**CLPB1**	Atp-dependent clp protease atp-binding subunit clpb
	comp_71_111536_c0_seq2	1.25	1.29	*CYP22*	Cyclophilin-like peptidyl-prolyl cis-trans isomerase family protein
	comp_71_119064_c0_seq2	1.21	1.22	*CHY1*	beta-hydroxyisobutyryl-CoA hydrolase 1
**HP-retained -Regulators**	comp_159_133991_c3_seq1	1.54	3.30	DRIP2	E3 ubiquitin protein ligase DRIP2
	comp_159_108021_c0_seq1	1.44	1.58	**EIF2B**	Eukaryotic translation initiation factor 2 subunit beta
	comp_71_119665_c0_seq1	1.61	1.54	**F18K10.11**	probable U3 small nucleolar RNA-associated protein 7
	comp_159_126100_c0_seq12	1.32	1.50	*RH8*	DEAD-box ATP-dependent RNA helicase 8
	comp_159_124389_c0_seq2	1.24	1.34	EIF2A/IF2AH*	Eukaryotic translation initiation factor 2 alpha subunit
	comp_159_126718_c0_seq9	1.226771	2.88	*PIRL3*	Plant intracellular Ras-group-related LRR protein 3
**HP-induced -Effectors**	comp_159_117370_c2_seq34	1.17	3.24	**HSP17.6 C**	HSP20-like chaperones superfamily protein
	comp_71_125839_c0_seq24	1.04	2.54	*FTSH6*	ATP-dependent zinc metalloprotease FTSH6 6,chloroplastic
	comp_159_131813_c0_seq5	1.17	2.25	**HSP70-8**	Heat shock 70 kDa protein 8
	comp_159_120894_c0_seq1	1.10	2.22	**HSP22.0**	HSP20-like chaperones superfamily protein
	comp_71_121713_c0_seq6	1.00	2.14	**ATJ2**	Chaperone protein dnaJ 2
	comp_159_133734_c0_seq2	1.16	2.09	CLPB4*	Atp-dependent clp protease atp-binding subunit ClpB4
	comp_159_105638_c0_seq1	1.08	1.93	HSP70-2*	mediator of RNA polymerase II transcription subunit 37c
	comp_159_131037_c1_seq1	1.05	1.87	*ERDJ3A*	DNAJ heat shock N-terminal domain-containing protein
	comp_71_119211_c0_seq2	1.129688	1.80787	*BBD2*	Bifunctional nuclease 2
	comp_159_135145_c1_seq2	1.00	1.71	**HSP90-2**	heat shock protein 90 (HSP90) gene family
	comp_159_131326_c1_seq2	0.84	1.66	**ATJ3**	Chaperone protein dnaJ 3
	comp_71_121718_c0_seq3	0.935236	1.517909	*LON1*	Lon protease homolog 1
	comp_159_127038_c0_seq15	0.92	1.49	*NBP35*	Cytosolic Fe-S cluster assembly factor NBP35
	comp_71_114889_c0_seq3	0.901973	1.414939	*UREG*	Urease accessory protein G
**HP-induced -Regulators**	comp_71_109156_c0_seq1	1.040243	3.24288	*RAR1*	Cysteine and histidine-rich domain-containing protein RAR1
	comp_159_112673_c1_seq1	1.04	2.43	**HSFA3**	heat shock transcription factor a3
	comp_71_113010_c0_seq24	0.91	2.22	**HSFA1B**	Heat stress transcription factor A-1b
	comp_159_127883_c0_seq18	0.95	2.18	*TPR1-2*	Tetratricopeptide repeat (TPR)-like superfamily protein
	comp_71_122100_c0_seq5	0.939267	1.901021	*HDA19*	Histone deacetylase 19
	comp_159_125100_c2_seq3	1.14	1.87	RABA2b*	Ras-related protein RABA2b
	comp_159_122124_c0_seq2	1.048504	1.75	*GLP6*	Germin-like protein subfamily 1 member 13
	comp_71_113202_c0_seq2	0.992259	1.684136	*GLP1*	Germin-like protein subfamily 3 member 1
	comp_71_124444_c1_seq16	0.87	1.64	*GDPD6*	PLC-like phosphodiesterases superfamily protein
	comp_159_131509_c0_seq1	1.02	1.51	**BIP1**	Mediator of RNA polymerase II transcription subunit 37a
	comp_159_124485_c0_seq3	0.96	1.47	**T1O3.7**	Putative translation initiation factor eIF-1 A
	comp_71_124960_c0_seq4	0.85	1.34	**TIF3C1**	Eukaryotic translation initiation factor 3 subunit C
	comp_71_124744_c0_seq3	0.85	1.24	delta-ADR*	AP-3 complex subunit delta

Note: Protein names in bold represent Hub proteins, while GO-enriched proteins and KEGG-enriched proteins are underlined and marked with an asterisk, respectively.

HSMPs were detected in all 8 GO terms enriched in R4, with a total of 8 proteins. Among these, the BP category housed the majority of HSMPs, with“cell wall organization or biogenesis” enriched with the highest significance (Fig. 4**)**. 3 HSMPs exhibited up-regulation in GO terms: comp_159_125998_c0_seq1 (pectin methylesterase 31, PME31), comp_71_109193_c0_seq1 (S-formylglutathione hydrolase, SFGH) and comp_71_ 106493_c0_seq1 (homolog of bacterial cytokinesis Z-ring protein, FTSZ1). These proteins bolstered the cell wall, detoxified formaldehyde, and facilitated plastid division, evidencing ongoing intracellular repairs post heat stress. Notably, a down-regulated member of the RdDM (RNA-directed DNA methylation) pathway, FDM2, was reported in R4, indicating HP potentially modifying protein expression through suppressed gene silencing.

In S5, 55 GO terms were enriched, and 29 HSMPs, classified in 14 unique GO categories, were identified post de-redundancy. Noteworthy highly enriched GO terms included “PSII associated light-harvesting complex II catabolic process”, “cell morphogenesis involved in differentiation”, “photosystem II repair” and “heat shock protein binding”. The GO terms hosting most HSMPs were “hydrolase activity”, “response to heat”, “heat shock protein binding”, “peptidase activity“(Fig. 5**)**. HSMP functioning in “photosystem II repair”, LQY1 (comp_159_ 133513_c1_seq23), previously widely reported for reorganizing photosystem II under HS-induced high light conditions, was highlighted. Generally, HSMPs intersecting “response to heat” and “heat shock protein binding” were majorly molecular chaperones, e.g. HSP17.4B (comp_159_116978_c1_seq5), ATJ2/3 (comp_71_ 121713_c0_seq6, comp_159_131 326_c1_seq2), ClpB1(comp_71_123489_c0_seq2), ERDJ3A (comp_159_131037_ c1_seq1). While those under “hydrolase activity” and “peptidase activity” GO terms were predominantly proteases, e.g., FTSH6 (comp_71_125839_c0_seq24), EGY3 (comp_159_132147_c0_seq1), DEGP8 (comp_ 71_118005_c1_seq7). Three chief categories summarizing HSMPs were photosystem II repair, chaperonins, and proteases, mainly contributing to the restoration of protein homeostasis. Identified HSMPs localized in the chloroplast proposed an association with photosystem II heat resistance, with five specific proteins being notable: FTSH6, EGY3, DEP8, LQY1, LHCA4 (comp_159_119519_c1_seq1).

### KEGG enrichment analysis of DAPs

**Fig. 6:**
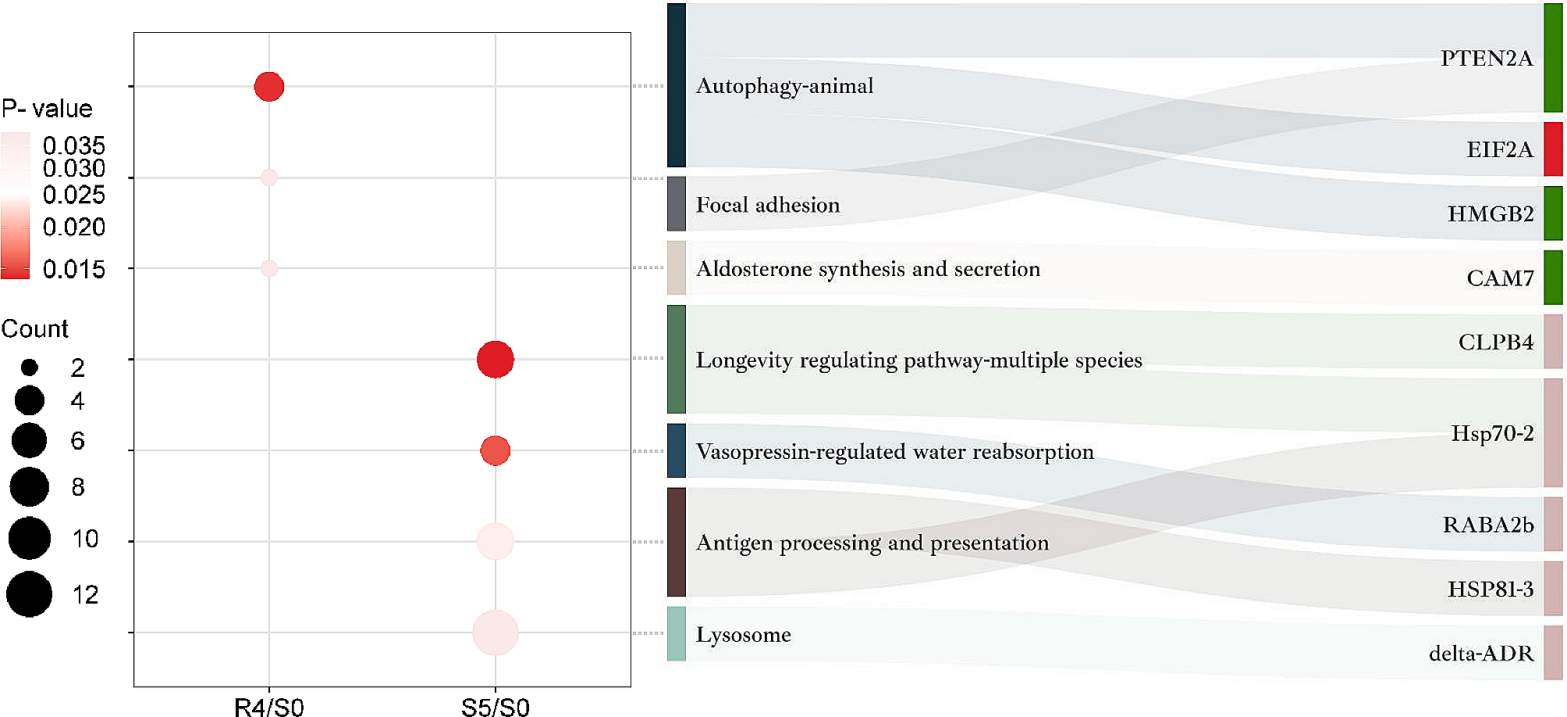
**KEGG enrichment analysis of DAPs.** The enriched KEGG pathways associated with DAPs in the R4/S0 and S5/S0 comparisons are delineated by the dots. The bubble plots solely display KEGG pathways containing HSMPs. The dot’s magnitude corresponds to the number of DAPs within the respective pathways, while their hue reflects the P-value from Fisher’s exact test. Intensifying red shades denote decreasing P-values, signifying increased statistical test significance. In addition, the adjacent Sankey diagram displays a clear correlation between the KEGG pathway nomenclature and their matching HSMPs in the same row. The regions shaded in deep red, deep green, and pale red following the protein names signify DAPs retained by HP, DAPs specific to R4, and DAPs induced by HP, respectively

3 and 7 KEGG pathways were found to be enriched in R4/S0 and S5/S0. Further details of the KEGG-enriched HSMPs are displayed in Table 1. In R4/S0, the pathways to possessing the HSMPs were “autophagy-animal” (3 HSMPs), “focal adhesion” (1 HSMPs), “aldosterone synthesis and secretion” (1 HSMPs), of which the “autophagy-animal” was most significantly enriched (Fig. 6**)**. The only upregulated HSMP identified in the “autophagy-animal” pathway was Eukaryotic translation initiation factor 2 alpha subunit, EIF2A (comp_159_124389_c0_seq2). The results indicate that continued protein translation during the recovery stage might be a key reason for the delayed degradation of HP-retained proteins, possibly preparing the plant for subsequent HS. Conversely, in S5/S0, the enriched KEGG pathway containing the HSMPs were “longevity regulating pathway-multiple species” (2 HSMPs), “vasopressin-regulated water reabsorption” (1 HSMPs), “antigen processing and presentation (2 HSMPs)”, “lysosome” (1 HSMPs) (Fig. 6**)**. The most significantly enriched pathway was “longevity regulating pathway-multiple species” and “vasopressin-regulated water reabsorption”. Analysis of HSMPs in these enriched KEGG pathway in S5/S0 revealed that proteins function in molecular chaperone (e.g., CLPB4, comp_159_133734_c0_seq2; Hsp70-2, comp_159_105638_c0_seq1; HSP81-3, comp_71_123670_c0_seq2) and vesicular transport and fusion (e.g., Ras-related protein, RABA2b, comp_159_125100_c2_seq3) provided the material basis for protein transport and metabolism at S5.

### Protein-protein interaction (PPI) networks among DEPs

**Fig. 7:**
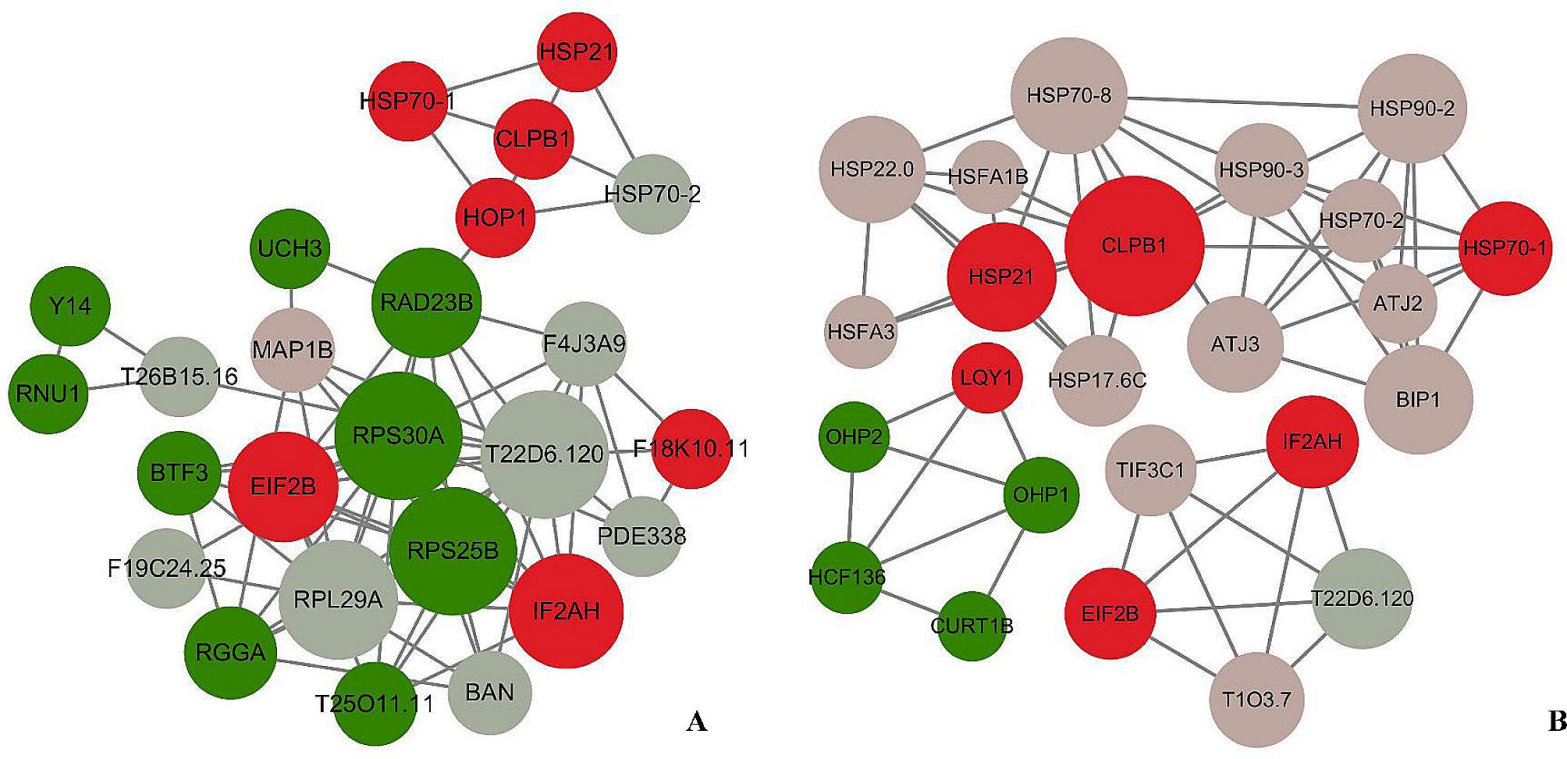
**Protein-protein interaction networks.** The interaction network of DAPs in R4/S0 (**A**) and S5/S0 (**B**) was analyzed and visualized via the STRING database and Cytoscape software (version 3.10.1). For the retrieval of the organisms, Arabidopsis thaliana was utilized. In this structure, only hub proteins representations are nodes, and any line between two nodes indicates an interaction. The larger the diameter of the circle, the higher the score in the PPI analysis. Dark green represented down- regulated R4-specific DAPs; Light green and dark red colors indicated down- and up-regulated DAPs retained by HP. Conversely, light red in A and B represents upregulated R4-specific DAPs and upregulated HP-induced DAPs, respectively. The minimum interaction score in R4/S0 and S5/S0 were was established at 0.4 and 0.7, respectively. More extensive information regarding nodes and proteins is available in **Additional file 5: Table ****S4**

PPI networks served to form testable hypotheses for predicting protein functions and pinpointing key regulators. To discern prime components related to HS memory, a PPI network was developed referencing the STRING database (http://string-db.org) utilizing Cytoscape software 3.10.1. PPI analysis was separately carried out at two different stages (R4, S5) (Fig. 7**)**.

With a moderate level of confidence (interaction score > 0.4), 258 differentially abundant proteins (DAPs) consisting of 123 R4-specific proteins and 135 heat pressure (HP)-retained proteins were incorporated to establish the PPI network at the R4 stage, including 80 nodes and 175 edges (**Additional file5: Table ****S4**. 25 proteins were singled out as hub proteins, with 8 DAPs up-regulated and 17 down-regulated **(**Fig. 7; Table 1**)**. Up-regulated DAPs were primarily linked to protein translation initiation (e.g., Eukaryotic translation initiation factor 2 alpha subunit, IF2AH_ARATH, comp_159_124389_c0_seq2; Eukaryotic translation initiation factor 2 subunit beta, EIF2B, comp_159_108021_c0_seq1) and functioned as molecular chaperones(e.g., CLPB1, comp_71_123489_c0_seq2; HSP70-1, comp_159_125549_ c1_seq5; HSP21, comp_71_109284_c0_seq3; Stress-induced- phosphoprotein1, HOP1, comp_ 159_127534_c1_seq6). Intriguingly, all of these were HP-retained proteins interacting with RAD23B (Rad23 UV excision repair protein family, comp_71_118763_c0_seq5) in the network, a down-regulated ubiquitin receptor. Among the down-regulated DAPs, including ribosomal proteins (e.g., H/ACA ribonucleoprotein complex subunit 2-like protein, T22D6.120, comp_71_105452_c0_seq1; Ribosomal protein S30 family protein, RPS30A, comp_159_131602_c0_seq8; Ribosomal protein S25 family protein, RPS25B, comp_159_115698_c0_seq3) were at the core of the network. Thus, it is reasonable to infer ribosome destruction led to most protein suppression in R4. HS memory-related proteins persisted, owing to a hampered ubiquitination pathway. Overall, changes in protein abundance at R4 majorly relied on translational and post-translational regulation.

At the S5 stage, the PPI network was built from 401 DAPs (135 HP-retained proteins and 266 HP-induced proteins) with high confidence (interaction score > 0.7) and composed of 77 nodes and 190 edges (**Table ****S4**). 24 proteins were identified as the hub proteins (Fig. 7; Table 1), featuring 5 down-regulated and 19 up-regulated DAPs. Most down-regulated proteins were photosystem-associated subunits, suggesting HS severely hindered the photosystem’s stability. Up-regulated proteins primarily consisted of HP-retained proteins, comparable to those seen at R4. Notably, two chloroplastic proteins, HSP21 and LQY1, were reported to play crucial roles in maintaining photosystem activity against HS. Furthermore, 13 HP-induced proteins were largely the HSF-HSP regulatory axis members. The ones that were induced in greater folds by HP were HSFs (e.g., HSFA1B/HSFA6A, comp_71_113010_c0_ seq24; HSFA3, comp_159_112673_c1_ seq1), sHSPs (e.g., HSP17.6 C, comp_ 159_117370_c2_seq34; HSP22.0, comp_159 _120894_c0_seq1) and dnaJ proteins (e.g., ATJ2/3, comp_71_121713_c0_seq6/ comp_159_131326_c1_seq2). It appears that the protein abundance shift at S5 was determined by transcriptional, translational and post-translational levels of regulation.

### RNA expression levels of heat stress memory-related genes

**Fig. 8:**
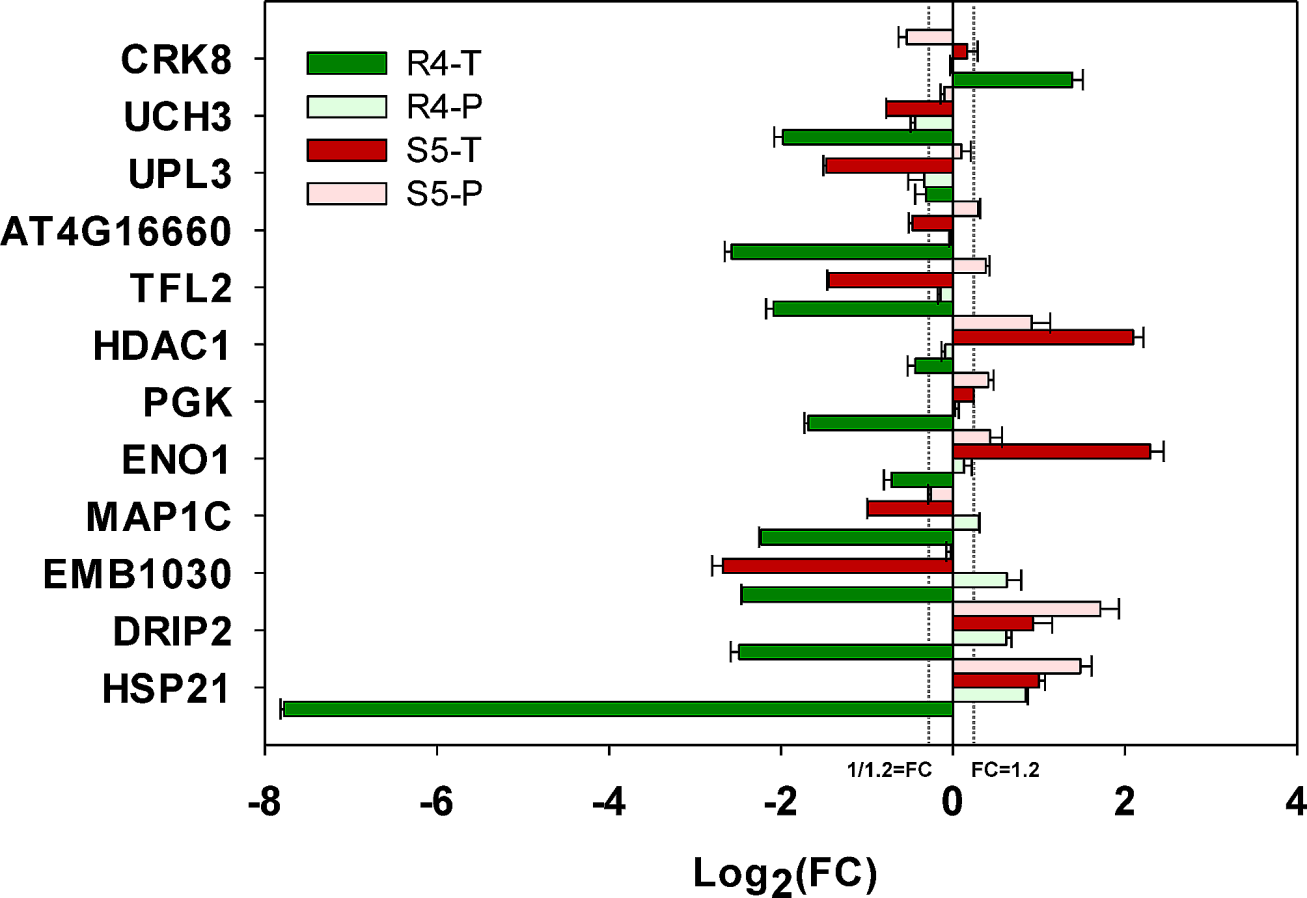
**RT-qPCR validation.** The comparative analysis was conducted on the protein levels of 12 selected DAPs and the transcript levels of their respective encoding genes. They were 4 R4-specific proteins, 6 HP-induced proteins and 2 HP-retained proteins that were randomly chosen. *ACTIN3* was chosen as a reference gene and the corresponding primer sequences are provided in **Table ****S6****.** Statistical differences between the transcript and protein levels of the representative genes at the two time points were compared separately using independent-samples t-tests with three biological replicates for each sample. Abbreviations: Log_2_FC, the logarithm to base 2 for Fold Changes (FC); CRK8, Cysteine-rich receptor-like protein kinase 8; UCH3, Ubiquitin C-terminal hydrolase 3; UPL3, Ubiquitin-protein ligase 3; AT4G16660, Heat shock 70 kDa protein 17; TFL2, Protein TERMINAL FLOWER 2; HDAC1, Histone deacetylase 19; PGK, phosphoglycerate kinase; ENO1, Phosphoenolpyruvate enolase1; MAP1C, Methionine aminopeptidase 1 C; EMB1030, EMBRYO DEFECTIVE 1030; DRIP2, DREB2A-interacting protein 2; HSP21, Heat shock protein 21

To examine expression patterns of DAPs determined from iTRAQ, we selected 12 representative DAP encoding genes for RT-qPCR analysis using specific primers. (Fig. 8, **Additional file 6: Table ****S5**, **Fig ****S2**). The selection process was executed carefully, prioritizing genes associated with specific biological pathways based on a thorough literature review and preliminary data analysis, such as UPL3 and HSP21. Additionally, we considered differential expression profiles from sequencing data to enhance the reliability and comprehensiveness of our findings, and a fraction of these genes were selected randomly. The selected genes encode for 4 R4-specific proteins (UCH3, UPL3, MAP1C, EMB1030), 6 HP-induced proteins (CRK8, AT4G16660, TFL2, HDAC1, PGK, ENO1) and 2 HP-retained proteins (DRIP2, HSP21).

The results showed that, at stage S5, the transcript levels of 8 out of the 12 selected genes correlated with observed fluctuations in their associated protein levels. Specifically, among 8 DAPs, coherence between transcript fluctuations and corresponding protein adjustments was observed in five genes (HSP21, DRIP2, ENO1, PGK, HDAC1). Conversely, at stage R4, except CRK8, transcript levels of the remaining 11 genes were down-regulated, despite an observed increase in the protein abundance for 6 genes. Among 6 DAPs, only UCH3 and UPL3 exhibited changes in transcript levels consistent with alterations in their associated protein levels. Notably, 11 genes exhibited significant variances between transcript and protein levels at both time points as per the independent-samples T-test, excluding UPL3 at R4/S0. In conclusion, compared to S5 (4/7, 57.14%), randomly selected DAPs in R4 stage (1/5, 20.00%) showed a smaller proportion of synchronized changes between transcript levels and their associated protein levels. This supports the notion that protein abundance regulation in R4 primarily occurs at post-transcriptional levels (e.g., protein translation enhancement or protein degradation inhibition), while in S5, transcriptional level regulation is preferred.

## Discussion

The sixth IPCC assessment r suggests an imminent rise in global temperatures by 1.5 °C to 2 °C over the forthcoming two decades [43]. As a consequence of global warming, there has been an observed increase in the occurrence of extreme high temperatures, which gravely impacts crop production [44]. The globally distributed cool-season grass species, tall fescue, which optimally grows within 18–24 °C, is frequently subjected to recurring heat stress. This positions it as a perfect candidate for investigating the core mechanisms of heat stress memory, while aiding the genetic improvement of grasses and crops for better heat tolerance. Nevertheless, due to the intricately heteropolyploid genetic composition of this species, proteomic research into heat stress memory remains limited, and only a handful of researchers have addressed this matter. In a study by Wang et al., (2020) [45], proteomic research was conducted with two days-interval in Azalea (*Rhododendron hainanense* Merr.) leaves. In contrast to Wang et al., (2020)’s methodology, this study introduced a twenty-hour interval or memory period, hoping to obtain elements that are more upstream in heat stress memory events. Combined with soft clustering, GO/KEGG enrichment, and PPI analysis, the key proteins and their potential regulatory networks were identified and elucidated.

### Tall fescue response to heat priming at physiological levels

Three components of the photosynthetic process: the oxygen-evolving complex (OEC) and photosystem II (PSII), carbon assimilation by Rubisco, and the ATP generation system, exhibit heat sensitivity [34]. In an effort to evaluate the structural and functional impacts of heat stress on the photosynthetic apparatus of tall fescue, the multistage (O-J-I-P) chlorophyll a fluorescence kinetic was observed and transmuted into measurable photosynthetic parameters using the JIP test. Previous research by our team demonstrated that mild heat stress pretreatment (referred as heat priming or HP) endows plants the capacity to endure lethal high temperatures [46, 47]. This was evinced by the stabilisation of PS II photochemical activities and the soluble protein content in leaves. The current study substantiates that HP incites a decrease in L-band (HP34) and M0 during the O-K phase. The data implies an augmented energetic connectivity between the reaction center and OEC in PSII [48]. Furthermore, it was noted that in leaves primed under triggering stress, FV and φE0 were elevated whilst ABS/RC and TR0/RC decreased in comparison to unprimed leaves. Comparable behavioural patterns were observed in leaves of chickpea (*Cicer arietinum* L.) and wheat (*Triticum sp*. L.) subjected to the HP procedure [49, 50], despite the variations in priming and triggering temperatures. HP appears to guide PSII towards initiating non-photochemical quenching to mitigate damage from excess light energy, thus ensuring the security of photosynthetic electron flow; this is further supported by the phenotype in Fig. 1b. The present study seeks to further explore the physiological and molecular mechanisms underpinning the enhanced heat resistance imparted by HP, by identifying and analyzing differentially abundant proteins in tall fescue leaves at S0 (control), R4 (primed), and S5 (triggering) time points employing TMT-labeling comparative proteomics.

### Effectors which confer HS memory

In the current study, we identified 135 HP-retained proteins, of which only 45 were reinvigorated by heat stress (HS). Their expression profile resembles that of Type I HS memory proteins, which can persist for an extended period without immediate disappearance post-HS [16]. This reprogramming of intracellular proteins serves to equip plants with increased resistance to lethal HS.

Our analysis focused primarily on differentially-expressed proteins (DEPs) localised in chloroplasts when discussing the impact of HS memory on photosynthesis. The chloroplast, a semi-autonomous organelle of endosymbiotic origin, contains between 2500 and 3000 proteins, of which over 95% are encoded by nuclear genes. Following translation at cytoplasmic ribosomes, preproteins are imported via the TOC-TIC translocator [51]. Nevertheless, in their unfolded state during import and internal sorting, these proteins are susceptible to misfolding and nonspecific aggregation at high temperatures, which can result in subpar photosynthetic capacity and cytotoxicity. To maintain chloroplast homeostasis, protein quality control (PQC) networks, including modules like molecular chaperones, intrinsic proteases, the ubiquitin-proteasome system (UPS) and autophagy, adjust protein levels in a timely manner [52, 53]. In this study, we identified 7 accumulated chloroplastic DEPs at R4, namely DJC77, HSP21, DEGP8, EGY3, LQY1, PsbR and LHCA4. These proteins function within the PQC as chaperone proteins, proteases and photosystem assembly factors.

Cytosolic chaperones systems (HSP90/HOP, HSP70/14-3-3) and chloroplast stromal chaperones (HSP93, cpHsp70, and Hsp90C) were respectively implicated to drive the preproteins arrive and across the chloroplast envelope membrane, through associating with N-terminal transit signals [54–56]. Heat stress can disrupt this process, modifying the role of chaperonins from transporters to facilitators of proteolysis [57]. Specifically, cpHSP70-1, in conjunction with GUN1 (GENOMES UNCOUPLED 1) and CLPC1, links the CLPP protease to TOC channels, thereby contributing to chloroplast protein quality control systems. In another mechanism, HSP70-4 cooperates with CHIP (Carboxy-terminal Hsp70-Interacting Protein) to expedite the UPS-mediated degradation of preproteins in the cytoplasm [58]. Further, in collaboration with sHSP, HSP70, and 26s proteasome subunits, HSP101/CLPB1 synchronizes the breakdown and hydrolysis of protein aggregates under heat stress [59]. In stressed granules, HSP101 safeguards eukaryotic translation elongation factor (eEF1B) and eukaryotic translation initiation factor 4 A (TIF4A), facilitating the release of ribosomal RNA during recovery [60, 61]. Notably, HSP101/CLPB1 may extend heat stress memory through a positive feedback loop with HSA32 [62]. The ability to retain HOP1, HSP70-1, and CLPB1 can bolster resistance to the unfolded protein response and efficiently manage the translation machinery in the cytoplasm. Additionally, heat-induced perturbation of preprotein import can destabilize multiprotein complexes in the chloroplast stroma or thylakoid, potentially triggering chloroplast UPR due to protein accumulation [63]. LQY1-HHL1 has been connected to the repair of photodamaged PSII under high light conditions by controlling the restoration and reorganization of the PSII complex [64]. Furthermore, PsbR is necessary for the stable assembly of the oxygen-evolving complex protein PsbP in the PSII core complex, and it collaborates with PsbQ to optimize photosynthetic water splitting and electron transfer [65, 66].

It is also important to note the collaboration between the photosystem supramolecular complex and chloroplastic sHSP, which forms the first line of defense against UPR [42, 47]. Overexpression of CPsHSPs often minimizes oxidative injury to the photosynthetic apparatus, thereby increasing its photochemical activity under heat stress [67]. In our study, we found that the chloroplast protein with the highest abundance at R4 was HSP21, an Arabidopsis HSP21 homolog known as a key component of heat stress memory [68]. We also observed accumulation of the internal plastid protease DEGP8. Located in the thylakoid lumen, DEGP8 forms a heterocomplex and associates with several thylakoid proteins, contributing to the turnover and repair of damaged PSII [69]. Interestingly, EGY3, an enzyme lacking protease activity, was also present at R4. It is induced by high temperature and light [70]. Prior research shows that EGY3 stabilizes CSD2, thereby regulating chloroplast ROS homeostasis and promoting retrograde signaling. We also observed a reduction in RAF1.1 in egy3 mutants [71], suggesting its potential role in Rubisco assembly stabilization. In addition, we found that RBCX1, a chaperone involved in the RuBisCO assembly process, was also enriched at R4. Taken together, these results reveal a complex game between chaperonins and proteases in determining the fate of proteins. They largely maintain protein homeostasis in chloroplasts during recurring heat stress. As these defenses need timely generation and the spatial distances limit direct communication between the nucleus and chloroplasts, it becomes especially necessary to retain these proteins over a period of time.

In this study, we identified 266 heat pretreatment (HP)-induced proteins at the S5 stage, postulated to possess Type II memory [16]. Although this categorization method might not swiftly designate memory proteins, the steep rise of these protein under heat stress (HS) boosts our confidence. We concentrated specifically on hub DAPs at S5 using Protein-Protein Interaction (PPI) analysis, finding, as expected, that most were Heat Shock Proteins (HSPs). As a universally preserved buffer system combatting protein misfolding and aggregation, HSPs are deeply entwined in various abiotic stresses [72]. These proteins are grouped into six families according to molecular weight: HSP100, HSP90, HSP70, HSP60, HSP40 (J protein), and sHSP. The HSP100 first dissolves aggregated proteins which are then forwarded to the HSP70 system for refolding, reprocessing, until they eventually revert to functionally normal proteins with the assistance of HSP60 [73]. Here, J protein acts as a co-chaperone for HSP70 to augment the ATPase activity of HSP70 [74]. Meanwhile, sHSPs bind to unnatural proteins, precluding irreversible aggregation, and these complexes are ultimately processed by HSP70/HSP100 [75]. HSP100s serve dual roles: not only as chaperone proteins but also as proteases, effectively determining the ultimate fate of the protein substrate. This study found CLPB1/HSP101, a renowned memory effector, to be central in the PPI analysis of both R4 and S5. We also noticed a chloroplast-localized CLPB3 strongly stimulated by HP at the S5 stage. Recent research suggests a potential role for CLPB3 in disentangling protein aggregates from the thylakoid membrane [76]. Hsp90 is significant as most of its interacting substrates are signaling proteins. For example, the Heat Shock Factors (HSFs) are docked by HSP70/90 under typical conditions, whereas they are released to respond to HS due to a fiercer competition with client proteins [11]. Besides, HSP90.1 partners with ROF1 and HSFA2, subsequently, this heterotrimer relocates to the nucleus after an HS encounter, inducing continuous HSP production, and extending HS memory during the recovery period. Conversely, an NBR1 accumulation during recovery mediates the degradation of HSP90.1 and ROF1 via autophagy, consequently wiping out the memory [20, 30]. The potential roles of AJ3 and HSP17.4b as memory effectors under HS were also substantiated. Earlier reports suggest that the farnesylated AJ3, in association with HSP70-4 and localizing in stress granules, obviates protein aggregation, thereby enhancing plant survival under sustained, moderate HS (37℃, 4d) [77]. Additionally, HSP17.4-CII functions as a corepressor of HSFA2 and boosts its deposition in tomato stress granules, whilst it solubilizes in the presence of HSP 17.4-CI [78]. This mechanism could potentially support the secure storage and release of HSFA2 amid recurrent HS events. In the current study, HSF17.4b, a homolog of tomato HSP17.4-CII, was present during R4.

### Underlying mechanisms in DAPs reprogramming by HS memory

Our data suggest that R4-specific proteins serve as coordinators of HSMPs as they do not appear to be immediately involved in the Heat Stress Response (HSR). This hypothesis is corroborated by our Protein-Protein Interaction (PPI) analysis results, which suggest a link between the maintenance of HP-retained proteins and a regulatory network of ubiquitination, revolving around UPL3, RAD23B, and UCH3. UPS-mediated protein degradation consists of two stages, the first stage is the attachment of ubiquitin molecules to substrate proteins, and the second is the degradation of modified-substrate by the 26 S proteasome. The transfer of ubiquitin molecules requires the participation of ubiquitin activating enzyme E1, ubiquitin conjugating enzyme E2, and ubiquitin ligase E3. First the cysteine residue (Cys) of E1 covalently binds to the terminal glycine (Gly) of the ubiquitin molecule via a high-energy thioester bond, in which requires ATP consumption. Then the ubiquitin molecule is transferred to the Cys residue of E2 to form the E2-ubiquitin thioester complex, and finally E3 transfers the ubiquitin molecule from E2 to the substrate protein [79]. The specific role that E3 plays in recognizing target substrate explains the complexity and volume of genes encoding E3 in the plant genome. [80].

In our study, we identified a significantly down-regulated HECT (Homologous to the E6-AP Carboxyl Terminus)-type ubiquitin ligase in R4, UPL3. UPL3, previously recognized for its pleiotropic regulatory roles, influences elements such as flavonoid synthesis, seed maturation, hormone-induced development, and stress response, by targeting related transcription factors [81–83]. We report the presence of the ethylene-responsive component, Hevein-like preproprotein (HEL) [84], in R4, which aligns with prior research findings that highlighted UPL3’s role in shutting down the EIN3-induced transcriptional cascade. Similarly, the down-regulation of UPL3 should restore the NPR1-mediated plant immune response. In addition, we characterized differing ubiquitin conjugates in UPL3 mutants and found an enrichment of ubiquitin levels for enzymes involved in the Calvin-Benson cycle and a decrease for enzymes in carbon metabolism. In addition, differential ubiquitin conjugates were characterized in *upl3* mutants [85], which showed the ubiquitin levels were enriched for enzymes in Calvin-Benson cycle, e.g., ribulose-1,5-P_2_-carboxylase (RuBisCO), phosphoglycerate kinase (PGK), while reduced for enzymes in carbon metabolism, e.g., phosphoenolpyruvate carboxylase (PPC2) and hexokinase 1(HXK1). In combination with PPC2 and HXK1, UPL3 regulates their protein stability and leads to a reduction in starch and sucrose accumulation. Intriguingly, UPL3 appears to target several HS memory related components, such as RAD23D, BRM, and ATX1 [85]. These components are instrumental in maintaining chromosomal accessibility and H3K4me3 during heat stress recovery [23, 27]. This binding facilitates the maintenance of chromosomal accessibility and H3K4me3 during heat stress recovery. In conclusion, the decrease of UPL3 during the interval stage could have activated a series of regulatory proteins that reconfigure the hormone response network and carbon metabolism pathways and sustain the chromatin opening of HS memory genes, a critical subset of effectors essential for cellular retention of acquired thermotolerance.

Proteins of the RAD23 (Radiation Sensitive23) type are members of the UBL-UBA (ubiquitin-like-ubiquitin-associated) shuttle family. The UBA domain at the C-terminal is believed to identify target proteins marked with polyubiquitin, whereas the UBL domain situated at the N-terminal functions to interact physically with proteasome receptors including Rpn1, Rpn10, and Rpn13. This assigns it the capacity to function as ubiquitin receptors and transporters in the ubiquitin-26 S proteasome system (UPS) [86]. There is extensive evidence that RAD23 plays a crucial role in multiple abiotic stress responses. Notably, interactions between MdRAD23D1 and MdPRP6 have been documented, which expedite the degradation of MdPRP6 by the 26 S proteasome, leading to accumulation of free proline that enhances drought resistance in apples [87]. Furthermore, a study by Hou et al. (2020) [88] indicates how the UBA domain of CsRAD23 collaborates with CsPNG1 in vitro, contributing to the endoplasmic reticulum-associated degradation pathway (ERAD). This suggests its potential influence over salt tolerance in cucumbers (*Cucumis sativus* L.).

UCH3 (Ubiquitin Carboxyl-terminal Hydrolase 3) is a deubiquitinating enzyme (DUB), associated with maintaining the circadian clock cycle’s regularity at high temperatures. Evidence from triple uch mutants of Arabidopsis demonstrate elongated circadian clock periods with increased TOC1(TIMING OF CAB EXPRESSION1) and GI (GIGANTEA) transcripts at 29 ℃ [89]. At 28 ℃, TOC1 undergoes degradation by ZTL (ZEITLUPE), facilitating thermomorphogenesis [90]. Significantly, ZTL, defined as an F-box E3 ubiquitin ligase, ensures protein quality control and maintains the stability of the circadian clock under heat stress (HS), with the aid of co-chaperone protein HSP90 and GI [91]. These form a ternary complex to facilitate mutual maturity [92]. We posit that UCH3 helps regulate TOC1 abundance alongside ZTL through possible intertwined mechanisms, perhaps antagonistically. Conversely, the intercommunication between responses to HS and circadian clock networks is well documented. For example, HSFA3, a target of CCA1 (CIRCADIAN CLOCK-ASSOCIATED 1), is one such case [93]. Interestingly, the expression of Heat Shock Response (HSR) genes exhibits circadian characteristics. In a heat stress environment, principal regulators of acquired thermotolerance, like HSFA2, are expressed throughout the day. In contrast, downstream genes such as HSP21, APX2, and HSFA32 tend to be highly induced during dawn than evening [94]. These genes seem to exhibit readiness in the morning to mitigate potential damage from increased light and temperature. It’s essential to note that all these are HS memory genes, and components of the circadian clock could be implicated in building HS memories. These findings suggest a possibility whereby UCH3 operates as a regulator of HS memory using CCA1-TOC1 oscillations.

In our study of HP-retained proteins, we similarly recognized an E3 ubiquitin ligase, DRIP2 (DREB2A-INTERACTING PROTEIN2), that identifies DREB2A (DEHYDRATION-RESPONSIVE ELEMENT BINDING PROTEIN2A) as its client. Known as a cross-regulator of drought and heat stress [95], DREB2A’s protein stability is tightly monitored and rapidly degraded via the Ubiquitin Proteasome System (UPS) under standard temperatures. Apart from DRIP2, DRIP1 and BPM2 (BTB/POZ AND MATHDOMAIN 2) have also been reported as E3 ubiquitin ligases involved in UPS [8, 96]. However, our results indicate that DRIP2 was preserved by HP at the R4 stage and transferred to the S5 stage. This suggests that the activity of DREB2A is managed by a more complex network. In fact, earlier studies have shown that the stability of DREB2A protein under heat stress is jointly adjusted by phosphorylation and SUMOylation, both acting through DREB2A’s negative regulatory domain. While phosphorylation promotes DREB2A degradation, SUMOylation preserves it [97, 98]. The role of DRIP2 in mediating the SUMOylation of DREB2A, contributing to heat shock memory, remains to be determined.

The transcriptional regulatory mechanisms of HSMPs have been exhaustively researched, primarily through HSFA2/HSFA3 heteromeric complexes, which notably recruit transcriptional co-activators and histone H3K4 methyltransferases [18]. The trimethylation of histone H3 lysine 4 (H3K4me3) is the predominant mark during HS memory, associated with the transcriptional activation of genes [15]. To accomplish the establishment and removal of specific histone methylation modifications, organisms have developed a variety of enzymes. These enzymes include three classes of proteins: readers, writers, and erasers. This study identified various H3K4me3 readers (e.g., AL1, AL5, and AL6) and H3K9me erasers (JMJ25) within HS-induced proteins. Nevertheless, only the protein abundances of AL1 and JMJ25 exceeded 1.44 in S5/R4. Alfin-like proteins (e.g., AL5, AL6) are thought to localize in the nucleus and facilitate plant adaptation to salt and drought stress [99]. Subsequent research revealed that AL1 directly binds to the promoters of negative regulator genes in ABA signaling, suppressing their expression (e.g., GRF7), which results in the activation of ABA/stress-responsive genes (notably, DREB2A) [100]. We hypothesize that ALs could potentially enhance HS resistance. However, H3K9me2, an epigenetic marker associated with transcriptional inactivation, is closely linked with DNA methylation and inversely related to H3K4me3 in plants. JMJ25/IBM1 is implicated in removing H3K9me1/2, thus preventing the coupling of H3K9me2 and DNA methylation, which protects genes from silencing [101]. Although ALs and JMJ25 could potentially regulate HS resistance, their precise roles in HS memory remain unclear and necessitate further investigation.

## Conclusion

Our investigation has underscored the survival advantage conferred by pre-treating plants with HP under conditions of lethal heat stress, marked by an augmented electron transfer efficiency within PSII. Through a comparative proteomic analysis of tall fescue leaves across distinct stages (S0, R4, and S5), we have delineated the potential mechanisms underlying the enhancement of PSII photochemical activity facilitated by HS memory. A total of 526 differentially abundant proteins (DAPs) were delineated as HSMPs. GO and KEGG enrichment analyses have delineated that HSMPs were predominantly associated with the “autophagy pathway” in R4 and with “PSII repair”, “HSP binding”, and “peptidase activity” in S5. 7 chloroplast-localized HSMPs (HSP21, DJC77, EGY3, LHCA4, LQY1, PSBR and DEGP8, R4/S0 > 1.2, S5/S0 > 1.2) have been identified as effectors intricately linked to PSII heat stress memory, which was mostly classified in cluster 4. Protein-protein interaction (PPI) analysis has suggested that the ubiquitin proteasome system, centered on UPL3, RAD23b, and UCH3, could potentially account for the selective retention of memory effectors in R4. Lastly, we conducted RT-qPCR validation on 12 genes, revealing that, relative to S5, R4 exhibited diminished consistency between the transcript and protein levels, further bolstering the concept of post-transcriptional regulation of HP-retained proteins in R4. Our findings furnish novel insights into the establishment of HS memory under recurring high temperature episodes and furnish a conceptual framework for the breeding of thermotolerant crops endowed with enhanced PSII functionality.

## Methods

### Plant materials and growth conditions

The current study employed the heat-resistant Tall fescue (*Festuca arundinacea* Schreb) genotype “TF71”, which showcased remarkable over-summering performance in Wuhan, China (N30°32′40.47″, E114°24′44.50″). The tillers deriving from the same plant were meticulously propagated in plastic pots, then grown in a controlled greenhouse environment that maintained natural light, a day/night temperature of 22/18°C, and an average relative humidity of 70%. Subsequently, the seedlings received bi-weekly fertilizer treatments with half-strength Hoagland’s solution (1/2 HS) and were mowed weekly to expedite tillering. Following a two-month establishment period, the plants and accompanying culture medium were relocated to growth chambers. Certain plants were shifted to the hydroponic system for physiological assays. All growth conditions were standardized to a 22/18°C daily temperature (day/night), a 14/10 h photoperiod, a photosynthetically active radiation (PAR) level of 220 w/m2, and a relative humidity of 70%.

### Treatments and experiment design

The uniform adapted monoclonal population was divided into four groups. “Priming” (P) groups were subjected to the meticulously designed heat priming protocol prior to experiencing a simulated heat wave. In details, after 7-day adaptation to growth chambers’ surrounding, plants were subjected to mild heat shock (34 °C) at noon (10:00 am-14:00 pm) to imitate temperature peak period in summer of Wuhan, China. This process was repeated four times to establish a robust ‘memory’ of previous environmental deviations within the plants. In P groups, HP40/HP34 were triggered the priming effect as 40 °C/34°C and sustained the temperature for 36 h. While ‘No-priming’ (N) groups directly suffered high temperature without priming process. In NP40/NP34 group, plants were kept in 22/18°C until 40 °C/34°C treatment at same time as P group. The change in temperature was accomplished by moving the plants into a pre-heated growth chamber set at the desired temperature.

### Physiology assays

We investigated the variances in the structure and operation of PSII under specific experimental conditions via a chlorophyll fluorescence transient analysis facilitated by a Pulse-Amplitude-Modulated (PAM) Chlorophyll Fluorometer (PAM2500, Heinz Walz GmbH). We irradiated fully expanded leaves of tall fescue with saturated light (650 nm, 3500 mmol m^− 2^ s^− 1^) following a 30-minute period of dark adaptation. Fluorescence signals at specified intervals (0.02 µs, 2 ms, 30 ms, etc.) recorded as the basic parameter, and these data were then utilized in a JIP-test to derive meaningful photochemical indicators according to the energy flux theory. We further calculated Wk= (F_t_− F_o_)/ (F_K_− F_o_) and deduced the fluctuation in energy connectivity within the PSII. ΔW_K_ = W_K treatment_ - W_K control_ to delineate the L-band (approximately 0.15 ms). The positive or negative L-band values, when compared to the control, signify a decrease or increase, respectively, in PSII energy connectivity. Each treatment was replicated five times, with details provided in **Table ****S1**. Finally, we photographed the phenotype one week post heat treatment procedure.

### Preparation of samples

#### Protein extraction

Only samples from HP40 group were carried out comparative proteomic analysis by Tandem mass tag (TMT) technology. The sampling time was set as 0 h (S0), 96 h (R4), 137 h (S5) on behalf of three consecutive phases: control before ‘heat priming’, multiple ‘heat priming’ stimulus and triggering stress, which were depicted in Fig. 1. We took leaves as research objects and each sample had three replicates. Entire leaves were instantly frozen in liquid nitrogen and grinded into powder with a pestle and mortar. Then the powder was sufficiently blend with 5 volumes trichloroacetic acid (TCA)/ acetone(1:9)using vortex mixer and incubated at -20 °C overnight. The mixture was centrifuged at 10,000 rpm for 40 min at 4 °C. After removing the supernatant, the precipitate was washed three times with pre-cooling acetone. The final pellet was complete dried and resuspended in lysis buffer consisting of 4%SDS, 100mM Tris-HCl, 1mM DTT. The samples were conducted ultrasonic disruption in a manner which has 10 cycles as follows: 10 s (80 W), 15 s (interval). After 14,000 g centrifugation for 40 min, the supernatant was filtered and collected to determine protein concentration by the BCA (bicinchoninic acid assay) method.

#### Trypsin digestion and peptide quantification

The comparative proteomic analysis was solely conducted on samples from the HP40 group, leveraging Tandem Mass Tag (TMT) technology. The study design included three distinct phases represented as: ‘control before heat priming’ (S0, 0 h), ‘multiple heat priming’ stimulus (R4, 96 h), and ‘triggering stress’ (S5, 137 h), as outlined in Fig. 1. Our research focused on leaves, each test condition having three replicates. We immediately froze each leaf in liquid nitrogen and finely ground them using a pestle and mortar. After which, the sample was thoroughly mixed with five volumes of trichloroacetic acid (TCA)/acetone (1:9) using a vortex mixer, and incubated at -20°C overnight. After centrifugation at 10,000 rpm for 40 minutes at 4°C, we removed the supernatant and washed the precipitate thrice with pre-cooled acetone. We then allowed the final pellet to dry completely before reconstituting it in a lysis buffer composed of 4% SDS, 100mM Tris-HCl, and 1mM DTT. The samples underwent ultrasonic disruption through ten cycles of 10-second pulses at 80 W followed by a 15-second interval. After centrifugation at 14,000 g for 40 minutes, we filtered the supernatant and collected it for protein concentration determination via the Bicinchoninic Acid Assay (BCA) method.

#### TMT labeling and peptide fractionation

In this study, the Thermo Fisher Scientific TMT 10plex Isobaric Label Reagent was employed to label 100 µg of the 9 samples, which comprised three stages in the HP40 group, with three replicates each. The corresponding tags were 126 N, 127 N and 127 C for S0; 128 N, 128 C, 129 N for R4; and 129 C, 130 N, 130 C for S5. Post-labeling, the samples were fractionated into 15 segments using a Pierce high pH reversed-phase fractionation kit (Thermo scientific) and an increasing acetonitrile step-gradient elution procedure. Following this, the samples were sequentially desalted and lyophilized prior to the LC-MS/MS analysis.

#### LC-MS/MS analysis and protein identification

Each fraction was injected for nanoLC-MS/MS analysis. The peptide mixture was loaded onto a reverse phase trap column(Thermo Scientific Acclaim PepMap100, 100 μm*2 cm, nanoViper C18)that was linked to a C18-reversed phase analytical column (Thermo Scientific Easy Column, 10 cm long, 75 μm inner diameter, 3 μm resin) immersed in buffer A (0.1% Formic acid). Subsequently, they were separated using a linear gradient of buffer B (84% acetonitrile and 0.1% Formic acid) directed at a flow rate of 300 nl/min. LC-MS/MS analysis was conducted on a Q-Exactive mass spectrometer for 90 min. The mass spectrometer, operating in positive ion mode, scanned precursor ions within a range of 300–1800 m/z. Survey scans were acquired at a resolution of 70,000 at 200 m/z and resolution for HCD spectra was set to 35,000 at 200 m/z, and isolation width was 2 m/z. MASCOT engine (Matrix Science, London, UK; version 2.2), embedded in Proteome Discoverer 2.4, was used to search the raw data from the MS/MS spectra to identify and quantitatively analyse the library. A self-constructed database of tall fescue transcriptomes was employed. The proteomics data obtained through mass spectrometry were duly submitted to the esteemed ProteomeXchange Consortium (https://proteomecentral.proteomexchange.org) via the iProX partner repository [102, 103], and were assigned the dataset identifier PXD053448.

Moreover, proteins that showed a differential abundance (DAPs) were characterised by a value of *p* < 0.05 (Student t-test) and an absolute fold change (FC) value of more than 1.2 (elevated proteins: FC > 1.2; lessened proteins: FC < 0.83). In our study, we chose a fold change threshold of S5/R4 > 1.44 based on the premise that consistent upregulation of protein abundance induced by heat stress should be observed across consecutive time points, assuming that if a significant difference is detected at S5 compared to R4, similar changes would likely exist at an earlier time point (S4). Although protein abundance at S4 was not directly measured, we inferred from the hypothesis that if S4/R4 exceeds a certain threshold due to heat stress influence (e.g., > 1.2), more pronounced changes would be expected at S5, reflecting the accumulation of biological effects over time, characteristic of type II heat stress memory. Thus, we anticipate that S5 would exhibit a sustained and strengthened early response, leading us to establish a higher threshold (S5/R4 > 1.44, i.e., 1.2 multiplied by 1.2). This approach ensures robust and meaningful identification of changes, reflecting the cumulative effect of heat priming, while also enhancing sensitivity for detecting biologically relevant changes in protein abundance and reducing the risk of false positives.

#### Bioinformatic analysis

The cluster analysis of the normalized quantitative data on differentially abundant proteins (DAPs) was conducted using Cluster 3.0 software. The temporal dynamic characteristics of protein expression profiles were assessed using the Mfuzz package, enabling us to softly cluster proteins with similar patterns and inferring functional connections of DAPs [104]. Gene Ontology (GO, http://www.geneontology.org) functional annotation and categorization was performed using Blast2GO software [105], resulting in the classification of three ontologies: molecular function (MF), biological process (BP), and cellular component (CC). Data from KEGG Orthology (KO) was extracted from the online Kyoto Encyclopedia of Genes and Genomes (KEGG) database (http://www.genome.jp/kegg/) and subsequently mapped to pathways [106]. Fisher’s Exact Test (P-values < 0.05) was used to determine the significance of protein enrichment for each GO term or KEGG pathway. Protein-protein interaction networks, applicable to all DAPs, were constructed using the STRING database version 12.0 (https://cn.string-db.org). The acquired datasets were based on Arabidopsis thaliana, incorporating all interactions with confidence scores of at least 0.4 in R4 and 0.7 in S5. These interaction networks were visualized using Cytoscape software (version 3.9.1).

### RT-qPCR validation

The transcription levels of 12 representative DAP-encoding genes were measured using the real-time quantitative polymerase chain reaction (RT-qPCR). Initially, total RNA was extracted from 0.1 g of leaves using the FastPure Universal Plant Total RNA Isolation Kit (Vazyme, China), as per the instruction manual’s procedures. Following the genetic DNA digestion, the RNA was reverse transcribed into cDNA with the HiScript II 1st Strand cDNA Synthesis Kit (Vazyme, China). The qRT-PCR was subsequently executed using the ABI Quantstudio 6 Flex real-time PCR system (Applied Biosystems, Foster City, CA) and SYBR Green master mix with low Rox (Yeasen, China) in 20 µL reactions. The PCR process included the following temperature steps: 2 min at 50 °C, 10 min at 95 °C, and 40 cycles of 10 s at 95 °C, and 60 s at 60 °C. The *ACTIN3* gene was used as a reference to calculate relative fold-differences based on comparative cycle threshold (2 − ΔΔCt) values [107, 108]. The primer sequences for the assessed genes are presented in **Additional file6: Table ****S5****.** Each sample was duplicated three times and the independent-sample T-tests (*P* < 0.05) were employed to compare statistical differences between transcript and protein levels of representative genes in R4 or S5.

### Electronic supplementary material

Below is the link to the electronic supplementary material.

Supplementary Material 1

Supplementary Material 2

Supplementary Material 3

Supplementary Material 4

Supplementary Material 5

Supplementary Material 6

## Data Availability

The mass spectrometry proteomics data had been deposited to the ProteomeXchange Consortium with the dataset identifier PXD053448.
