# Supplementary material for: Comparative proteomics in tall fescue to reveal underlying mechanisms for improving Photosystem II thermotolerance during heat stress memory
Source: BMC Genomics. 2024 Jul 9;25:683. doi: 10.1186/s12864-024-10580-z (PMC11232258; doi:10.1186/s12864-024-10580-z)
Supplement: Supplementary file 1 — Supplementary Material 1 [file 12864_2024_10580_MOESM1_ESM.docx]

**Additional file1:**

**Table S1 The variation of fluorescence transient parameters of tall fescue after heat priming**

|  | **NP34** | **HP34** | **NP40** | **HP40** | **Definitions** |
| --- | --- | --- | --- | --- | --- |
| **Data extracted from the recorded fluorescence transient OJIP** | | | | | |
| **F_0_** | 0.83a | 0.80a | 0.87B* | 0.92A* | Fluorescence at time 20 μs after onset of actinic illumination |
| **F_m_** | 3.14a | 3.17a | 2.72B* | 2.92A* | Maximal recorded fluorescence intensity, at the peak P of OJIP |
| **F_k_** | 1.77a | 1.73b | 1.72B* | 1.76A* | Fluorescence value at 300 μs |
| **F_j_** | 1.84a | 1.80a | 1.72B* | 1.81A | Fluorescence value at the J-step (2 ms) of OJIP |
| **F_i_** | 2.63a | 2.61a | 2.30B* | 2.49A* | Fluorescence value at the I-step (30 ms) of OJIP |
| **Fluorescence parameters derived from the extracted data** | | | | | |
| **Area** | 79.11a | 83.35a | 71.28A* | 71.39A* | Total complementary area between the fluorescence induction curve and F=F_m_ |
| **F_v_** | 2.32a | 2.38a | 1.84B* | 2.00A* | Maximal variable fluorescence |
| **V_k_** | 0.41a | 0.39a | 0.46A* | 0.42B* | Relative variable fluorescence at k step |
| **V_j_** | 0.44a | 0.42a | 0.46A* | 0.45A | Relative variable fluorescence at J step |
| **V_i_** | 0.78a | 0.76a | 0.77A | 0.79A* | Relative variable fluorescence at I step |
| **M_0_** | 1.64a | 1.57a | 1.83A* | 1.69B* | Approximated initial slope (in ms−1) of the fluorescence transient |
| **S_m_** | 34.14a | 35.08a | 38.63A* | 35.72B | Normalized total complementary area above the O-J-I-P transient |
| **Ss** | 0.27a | 0.27a | 0.25B* | 0.26A | Normalized total complementary area corresponding only to the O-J phase |
| **N** | 127.15a | 131.15a | 155.17A* | 135.31B | Turnover number of Q_A_ reduction events between time 0 and tF_m_ |
| **Quantum yields and efficiencies** | | | | | |
| **φP_0_** | 0.74a | 0.75a | 0.68A* | 0.68A* | Maximum quantum yield of primary photochemistry (at t = 0) |
| **Ψ_0_** | 0.16a | 0.18a | 0.54A* | 0.55A* | Efficiency/probability that an electron moves further than Q_A_- |
| **φE_0_** | 0.41b | 0.43a | 0.37B* | 0.38A* | Quantum yield of electron transport (at t = 0) |
| **φD_0_** | 0.17a | 0.17a | 0.32A* | 0.32A* | Quantum yield (at t = 0) of energy dissipation (at t = 0) |
| **φR_0_** | 0.16a | 0.18a | 0.15A | 0.15A* | Quantum yield for reduction of end electron acceptors at the PSI acceptor side |
| **δR_0_** | 0.39a | 0.41a | 0.42A | 0.39A | Efficiency/probability with which an electron from the intersystem electron carriers moves to reduce end electron acceptors at the PSI acceptor side (RE) |
| **γ RC** | 0.17a | 0.17a | 0.14B | 0.15A | Probability that a PSII Chl molecule functions as RC |
| **RC/ABS** | 0.20a | 0.20a | 0.17B* | 0.18A* | Q_A_-reducing RCs per PSII antenna Chl (reciprocal of ABS/RC) |
| **Specific energy fluxes (per Q_A_-reducing PSII reaction center/RC)** | | | | | |
| **ABS/RC** | 5.06a | 4.99a | 5.93A* | 5.53B* | Absorption flux (of antenna Chls) per RC (at t=0) |
| **TR_0_/RC** | 3.73a | 3.74a | 4.02A* | 3.79B | Trapping flux (leading to Q_A_ reduction) per RC (at t=0) |
| **ET_0_/RC** | 2.09a | 2.17a | 2.19A | 2.10A | Electron transport flux (further than Q_A_−) per RC (at t=0) |
| **DI_0_/RC** | 1.33a | 1.25a | 1.91A* | 1.74B* | Dissipated energy flux per RC (at t=0) |
| **RE_0_/RC** | 0.82a | 0.89a | 0.91A | 0.81B* | Electron flux reducing end electron acceptors at the PSI acceptor side, per RC |
| **Phenomenological energy fluxes (per excited cross section/CS)** | | | | | |
| **RC/Cs_0_** | 2.42a | 0.64b | 2.74A | 1.40B* | Density of RCs (Q_A_-reducing PSII reaction centers) (at t=0) |
| **ABS/Cs_0_** | 12.23a | 3.23b | 16.23A* | 7.73B* | Absorption flux per CS, approximated by F_0_ (at t=0) |
| **TR0/Cs_0_** | 9.01a | 2.42b | 11.01A* | 5.29B* | Trapped energy flux per CS (at t=0) |
| **ET0/Cs_0_** | 5.05a | 1.41b | 6.00A* | 2.93B* | Electron transport flux per CS (at t = 0) |
| **DI0/Cs_0_** | 3.96a | 1.01b | 5.02A* | 2.37B* | Dissipated energy flux per CS (at t = 0) |
| **Performance indexes** | | | | | |
| **PI_ABS_** | 0.0094a | 0.0092a | 0.0091B | 0.0097A | Performance index (potential) for energy conservation from exciton to the reduction of intersystem electron acceptors |
| **PI_Total_** | 0.0022a | 0.0022a | 0.0022A | 0.0023A | Performance index (potential) for energy conservation from exciton to the reduction of PSI end acceptors |
| **PI_CS_** | 0.12a | 0.03b | 0.15A* | 0.07B* | Performance index on cross section basis |

Parameters were calculated according to the method reported in (Yusuf et al., 2010). During statistical difference analysis, one factor was fixed and the effect of the other factor was analyzed with the independent sample T-test (p < 0.05). The differences caused by heat priming were marked by letters (a/A, b/B), while differences due to temperatures were marked with asterisk (*).
