# Supplementary material for: Comparative proteomics in tall fescue to reveal underlying mechanisms for improving Photosystem II thermotolerance during heat stress memory
Source: BMC Genomics. 2024 Jul 9;25:683. doi: 10.1186/s12864-024-10580-z (PMC11232258; doi:10.1186/s12864-024-10580-z)
Supplement: Supplementary file 4 — Supplementary Material 4 [file 12864_2024_10580_MOESM4_ESM.docx]

**Figure S1 The hierarchical cluster analysis among S0, R4, S5 samples**


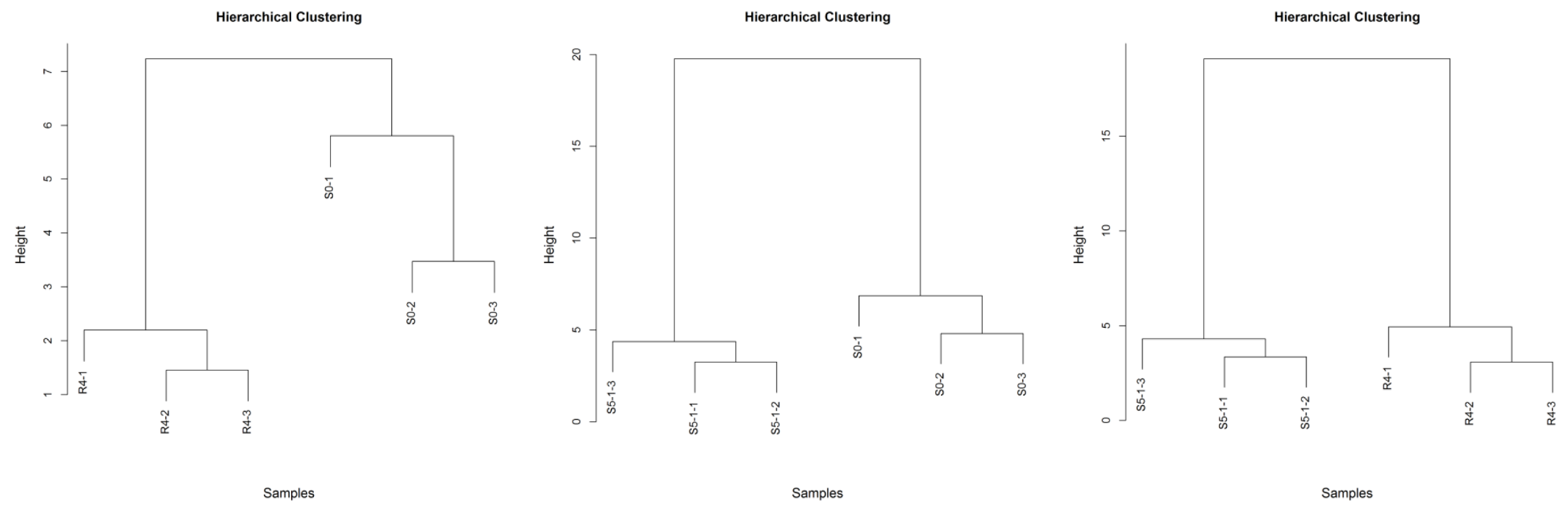


Samples sharing the same time were grouped together into distinct clusters. The clustering analysis revealed tight clustering of samples within each time, indicating high reproducibility and reliability of the proteomic data.
