# Supplementary material for: Comparative proteomics in tall fescue to reveal underlying mechanisms for improving Photosystem II thermotolerance during heat stress memory
Source: BMC Genomics. 2024 Jul 9;25:683. doi: 10.1186/s12864-024-10580-z (PMC11232258; doi:10.1186/s12864-024-10580-z)
Supplement: Supplementary file 5 — Supplementary Material 5 [file 12864_2024_10580_MOESM5_ESM.docx]

**Additional file 6:**

**Table S5 The primer for qRT-PCR**

| **Gene** | **Accession** |  | **Primer sequence (5 '-3')** | **Amplicon**  **Length(bp)** | **Melting temperature (℃)** | **GC Content(%)** | **Hairpin loop (bp)** | **Hydrogen bonds of the most stable dimer** |
| --- | --- | --- | --- | --- | --- | --- | --- | --- |
| *CRK8* | comp_71_58724_c0_seq1 | F | GCCAACCATGTCGACGATCAG | 179 | 81.53 | 57.1 | 0 | 8 |
|  |  | R | CGTTCGGAGACATTACCATCG |  |  | 52.4 | 0 |  |
| *UCH3* | comp_159_125448_c0_seq6 | F | GACTGTTGGCAATGCTTGTGG | 177 | 80.76 | 52.4 | 3 | 4 |
|  |  | R | TGAGCACCCTCCATCTCATCA |  |  | 52.4 | 4 |  |
| *UPL3* | comp_71_125826_c0_seq2 | F | GGTGGATGCTACTGTTAAGGC | 226 | 79.79 | 52.4 | 0 | 7 |
|  |  | R | CTCAAGTAGATTGACAATGGC |  |  | 42.9 | 4 |  |
| *AT4G16660* | comp_71_124950_c0_seq7 | F | CGGCTGGTCCTGGATCGATTC | 181 | 77.76 | 61.9 | 0 | 6 |
|  |  | R | GAGACAGTCAACATGTCCGTG |  |  | 52.4 | 4 |  |
| *TFL2* | comp_71_113852_c0_seq3 | F | CTGGAAGGTCTGGGAAGCCTG | 140 | 82.21 | 61.9 | 0 | 3 |
|  |  | R | CATGGTGGCGAAGTAGCGCAC |  |  | 61.9 | 3 |  |
| *HDAC1* | comp_71_122100_c0_seq5 | F | GTCCCTCCAAGCAAGTTCCTC | 99 | 82.63 | 57.1 | 0 | 6 |
|  |  | R | CTGGCAACTTGGTAGAGCTCC |  |  | 57.1 | 3 |  |
| *PGK* | comp_159_126402_c1_seq1 | F | CATCATTGGTGGCGGTGACTC | 108 | 85.06 | 57.1 | 0 | 5 |
|  |  | R | CCTTCCAGCAGCTCCAAGCTC |  |  | 61.9 | 4 |  |
| *ENO1* | comp_159_127115_c0_seq3 | F | CATTGCGGATCTAGCTGTTGG | 127 | 83.02 | 52.4 | 0 | 6 |
|  |  | R | GTACCGCACGTTGCCTAGCTC |  |  | 61.9 | 3 |  |
| *MAP1C* | comp_71_123536_c0_seq5 | F | CGGAGAGGCATGGATTCGGTG | 139 | 82.6 | 61.9 | 0 | 4 |
|  |  | R | CCGGAATGGGCAGAGTGATGC |  |  | 61.9 | 3 |  |
| *EMB1030* | comp_71_113460_c0_seq1 | F | TGGTTCAGAGACATCACAGGC | 163 | 80.78 | 61.9 | 0 | 4 |
|  |  | R | GATGTGTTGCATTGCTAATCC |  |  | 61.9 | 3 |  |
| *DRIP2* | comp_159_133991_c3_seq1 | F | GCATCATCCATCCAGAGGTAC | 173 | 86.43 | 52.4 | 0 | 6 |
|  |  | R | GAGGAGCCTACCGACGTCTGC |  |  | 66.7 | 4 |  |
| *HSP20* | comp_71_109284_c0_seq3 | F | CAGCAAGCTGTGGTATCCAATCTG | 152 | 85.7 | 50 | 3 | 5 |
|  |  | R | TGGTCAAGATTATCAGTGGACCCG |  |  | 50 | 4 |  |
| *ACTIN3* | comp_71_115894_c0_seq7 | F | CGGTAACATTGTGCTCAGTGG | 158 | 83.6 | 52.4 | 4 | 5 |
|  |  | R | CAAGGATGGACCCTCCGATCC |  |  | 61.9 | 4 |  |

**Figure S2 The melt curves of primer for qRT-PCR**


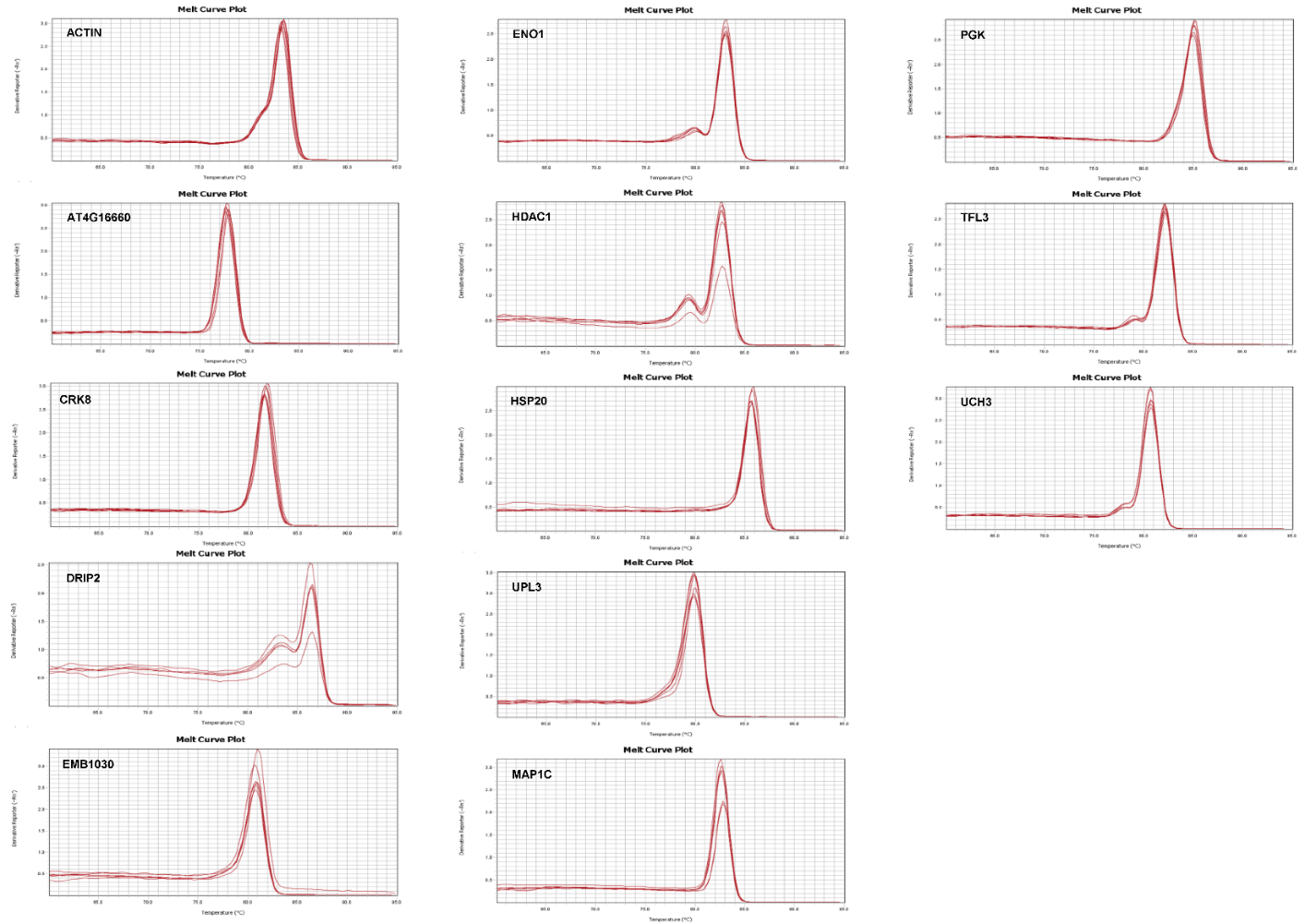


Each curve represents the melting profile of the amplified PCR product, with distinct peaks corresponding to specific amplicons. The analysis revealed sharp and single peaks for most target genes, indicating specific amplification and absence of non-specific products or primer dimers. These results demonstrate the robustness and reliability of the fluorescence qPCR data, validating its suitability for accurate quantification of gene expression levels in the experimental samples.
